# Genetic Engineering and Genome Editing for Improving Nitrogen Use Efficiency in Plants

**DOI:** 10.3390/cells10123303

**Published:** 2021-11-25

**Authors:** Vadim G. Lebedev, Anna A. Popova, Konstantin A. Shestibratov

**Affiliations:** 1 Forest Biotechnology Group, Branch of the Shemyakin-Ovchinnikov Institute of Bioorganic Chemistry of the Russian Academy of Sciences, 142290 Pushchino, Russia; schestibratov.k@yandex.ru; 2 Department of Botany and Plant Physiology, Voronezh State University of Forestry and Technologies named after G.F. Morozov, 394087 Voronezh, Russia; logachevaaa@rambler.ru

**Keywords:** nitrogen use efficiency, unintended effects, ammonium toxicity, phosphinothricin resistance, transgenic trees, genetically modified plants, nitrogen metabolism

## Abstract

Low nitrogen availability is one of the main limiting factors for plant growth and development, and high doses of N fertilizers are necessary to achieve high yields in agriculture. However, most N is not used by plants and pollutes the environment. This situation can be improved by enhancing the nitrogen use efficiency (NUE) in plants. NUE is a complex trait driven by multiple interactions between genetic and environmental factors, and its improvement requires a fundamental understanding of the key steps in plant N metabolism—uptake, assimilation, and remobilization. This review summarizes two decades of research into bioengineering modification of N metabolism to increase the biomass accumulation and yield in crops. The expression of structural and regulatory genes was most often altered using overexpression strategies, although RNAi and genome editing techniques were also used. Particular attention was paid to woody plants, which have great economic importance, play a crucial role in the ecosystems and have fundamental differences from herbaceous species. The review also considers the issue of unintended effects of transgenic plants with modified N metabolism, e.g., early flowering—a research topic which is currently receiving little attention. The future prospects of improving NUE in crops, essential for the development of sustainable agriculture, using various approaches and in the context of global climate change, are discussed.

## 1. Introduction

Nitrogen (N) is an essential nutrient for plant growth and development. As a component of proteins, enzymes, nucleic acids and plant growth regulators, this element is involved in many physiological and biochemical processes [1]. The double increase in the world grain production over four decades (1960–2000) was achieved due to a two-fold increase in grain yields owing to a seven-fold increase in the use of N fertilizers [2]. However, this had a high price. The world currently consumes about 115–120 million tons of N in fertilizers, and their production accounts for 1–2% of the world’s energy [3] and accounts for up to 50% of operational costs in agriculture [4]. Moreover, plants use as little as 30–50% of the applied N, depending on species and soil type, while the rest of it is lost in the environment [5]. As concerns the fate of the unused N in the environment, about one-third of it is denitrified to N_2_, one-third is washed out into groundwater as nitrates, and one-third escapes as N_2_O, an intermediate gas in denitrification [6]. The last two processes harm the environment: excessive nitrate levels in water cause algal blooms, which, in turn, eutrophicate natural water ecosystems and decrease their biodiversity [7], and the greenhouse effect of N_2_O is 300 times as significant as that of CO_2_ [8]. On the whole, annual economic losses from N pollution are calculated in hundreds of billions US dollars in the USA and Europe [9]. Therefore, it is important to increase the nitrogen use efficiency (NUE) of crops and, thus, preserve or even increase their productivity while reducing the use of N fertilizers. This will reduce both food cost and environmental pollution. Research in this field is carried out very intensively and the achievements are constantly summarized in reviews. Several excellent reviews have been published recently both on improving the NUE in general [10,11] and in specific plant species, mainly rice [12,13,14]. In our review, we focused on the practical achievements of bioengineering modification of key steps in N metabolism at the plant level of crops (biomass and yield) without taking into account model objects (Arabidopsis and tobacco). Particular attention was paid to woody plants as well as the unintended effects of modified N metabolism in transgenic plants.

## 2. Nitrogen Use Efficiency

The NUE is a very complex trait controlled by a large number of genes and environmental factors [15]. The term has several definitions based on different calculation methods and purposes. The choice of appropriate method to assess the NUE depends on the crop, its harvest product and the physiological process under study that is involved in the NUE [16]. As a concept, the NUE is expressed as a ratio of output (total plant N, biomass, grain yield) and input (total N, soil N or N-fertilizer applied) [17]. The use of N by plants has two main stages: uptake and utilization, and the latter can be further divided into assimilation and translocation/remobilization [17]. Therefore, most often, researchers use two terms: nitrogen uptake efficiency (NUpE), defined as the ability of plant to absorb N from soil, and nitrogen utilization efficiency (NUtE), defined as the ability to utilize the absorbed N to produce biomass or grains. Nitrogen uptake and utilization seem to be independently inherited traits; hence, favorable alleles can be combined by breeding to achieve a high NUE [18]. Generally, the NUtE is assessed separately for dicotyledonous and monocotyledonous species, because the final product is either biomass or grain, respectively [19]. However, this division is rather arbitrary: in many dicotyledonous species, the generative organs are the main component of the crop (for example, legumes), while for some monocotyledonous species, the biomass is the most important (for example, sugarcane). Improving the NUE is especially relevant to grain crops: they require large amounts of N fertilizers for maximum productivity while having an NUE as low as 33% [20]. Strategies developed to improve the NUE include the rational use of fertilizers, crop rotation and the use of plant varieties with improved the NUE that can more efficiently uptake nitrogen from the soil or utilize it. By changing the timing, doses and strategies of N fertilization, taking account of the phenological phases, soil and plant diagnosis, one can better synchronize N supply with the crop demand for N during the growth season. In China, for example, the average yields of rice, wheat and maize increased by 18.1%, 23.6% and 35.2%, respectively, due to integrated soil-crop system management without any increase in N fertilizer [21]. Inclusion of N-fixing crops in crop rotation and the use of slow-release fertilizers or nitrification inhibitors can also help reduce N fertilization while maintaining crop productivity.

Varieties with an improved NUE can be obtained by classical breeding as well as using biotechnological methods. Plant responsiveness to N availability depends on both genotype and the interaction of genotype with the N supply level [18]. Generally, with limited N supply, the NUE is higher than with an excessive one [15]. Most modern varieties were selected to grow on fertile soils and this may be a problem when they are used to improve the NUE for growing on soils with lower N levels. Interestingly, the genetically controlled variability of the NUE in the Arabidopsis accession collection did not depend on nitrogen supply levels, probably because no model plants had ever been selected for adaption to fertile soil, in contrast to crops [15]. A better understanding of the biology of N nutrition is a key to NUE improvement by genetic methods.

## 3. Nitrogen Metabolism in Plants

**Figure 1:**
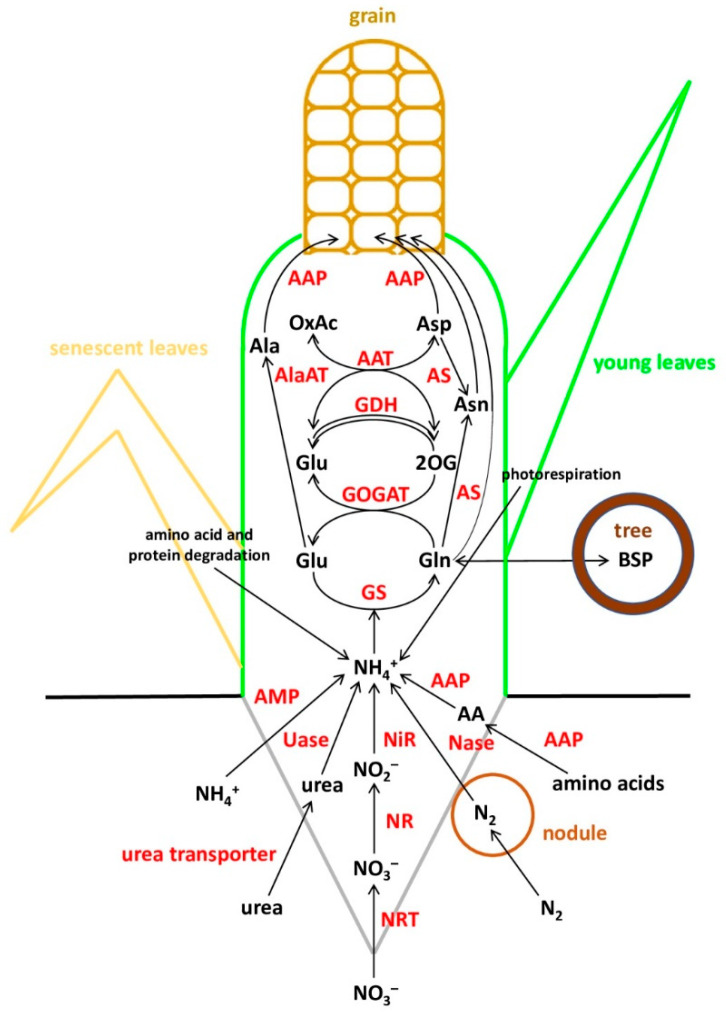
Schematic representation of nitrogen metabolism in plants.

For the sake of simplicity, in terms of nitrogen management, the life cycle of plants can be roughly divided into two main phases—vegetative growth and grain filling—tentatively separated by flowering [22]. In the first phase, young developing roots and leaves absorb and assimilate inorganic N to form amino acids for the synthesis of enzymes and proteins to be used mainly for plant growth and building up the photosynthetic apparatus. In the second phase, the accumulated N is remobilized: amino acids are released by protein hydrolysis and exported to the reproductive organs and storage organs (Figure 1). In reality, everything is much more complicated: N recycling can occur at early stages of development before flowering, photorespiratory ammonium is repeatedly assimilated and a considerable part of amino acids is released as a result of protein cleavage [22]. Additionally, some plants exhibit the stay-green phenotype, when the leaves retain their green color and photosynthesis capacity for a longer time after flowering [23], and the uptake of N during the generative stage can also be important, especially in perennial species.

### 3.1. Forms of Nitrogen

Plants can use various forms of N, primarily inorganic water-soluble nitrates (NO_3_^−^) and ammonium (NH_4_^+^), but also organic ones, such as amino acids, peptides and proteins. The availability of N in soil can strongly depend on factors such as precipitation, temperatures, soil type and pH [17]. Not only is the NO_3_^−^ concentration (1–5 mM) in a soil solution higher than that of NH_4_^+^ (20–200 µM), nitrate ions are more mobile and, therefore, more readily available to plant roots [24]. Nitrates are the main form of inorganic nitrogen in aerobic (cultivated) soils, whereas NH_4_^+^ is the main form of N on flooded and acidic soils [15]. However, in some soils, e.g., in boreal forest soils, organic nitrogen levels may be comparable with, or even exceed up to several times, those of inorganic nitrogen [25]. Urea, the world’s most widely-used nitrogen fertilizer, can also be directly used by plants, but its concentration in soil is even lower than that of NH_4_^+^ (10–70 µM) [26]. All these forms of nitrogen are absorbed by plants from soil either directly by roots or through the mycorrhizae. It is known that the form of nitrogen can also influence NUE [27]. For instance, greater leaf area and dry matter per unit N with NO_3_^−^ in comparison with other forms of nitrogen may be due to a better transport and assimilation of NO_3_^−^ in shoots. Thus, maximization of NUE requires understanding the impacts of different forms of N on cultivated plants. It should be also noted that legumes and some other plants can absorb atmospheric N_2_ owing to a symbiosis with bacteria in their root nodules.

### 3.2. Nitrogen Uptake and Transport

Four families of transmembrane proteins are involved in NO_3_^−^ absorption from soil solution and transport: nitrate transporter 1 (NRT1)/peptide transporter (PTR) family (NPF), NRT2, chloride channel (CLC) family, and slowly activating anion channel [3]. Best studied are proteins of the NRT1 and NRT2 families. Nitrate levels in soil may vary three to four orders of magnitude, and depending on NO_3_^−^ availability, plants use one of two different absorption systems: the high-affinity transport system (HATS) act at low NO_3_^−^ levels (<1 mM), while the low-affinity transport system (LATS) act at high (>1 mM) NO_3_^−^ levels [28]. Thus, these transporters ensure the uptake of NO_3_^−^ from soil in a wide concentration range. Most nitrate transporters from the NRT1 family belong to the LATS system, with the exception of some NRT1.1 proteins with both low and high affinity to nitrate. The representatives of the NRT2 family belong to the HATS system and act at low NO_3_^−^ levels [29]. In addition, some NRT1 members are peptide transporters. Two types of peptide transporters have been identified: PTRs are di/tripeptide transporters, while OPTs are tetra/pentapeptide transporters [30].

The uptake of NH_4_^+^, whose concentration in soil is normally below 1 mM, is mediated by ammonium transporters (AMTs) [31]. As concerns urea, only one high-affinity urea transporter was found in a variety of plants [26]. Although plants can absorb various forms of organic nitrogen, the main studies focus on amino acids. Amino acid transporters are currently divided into three large families: ATF or AAAP (amino acid transporter family, which includes eight subfamilies), APC (amino acid-polyamine-choline transporter family, which includes three subfamilies) and the newly identified UMAMIT (usually multiple acids move in and out transporters) family [32]. The best-studied amino acid transporters are the amino acid permease subfamily (AAP) of the ATF family.

### 3.3. Nitrogen Assimilation

Nitrate absorbed by the plant is reduced to NH_4_^+^. At first, NO_3_^−^ is converted to NO_2_^−^ by nitrate reductase (NR). The process occurs in the cytosol of root and shoot cells, with most plant species using NADH as reductant. The second stage takes place in plastids/chloroplasts: NO_2_^−^ is reduced to NH_4_^+^ by nitrite reductase (NiR), with ferredoxin (Fd) as reductant [27].

In higher plants, all ammonium—reduced from nitrates, absorbed directly from soil, released in photorespiration and other processes, and obtained by N_2_ fixation—is further assimilated into amino acids via reactions catalyzed by the glutamine synthetase/glutamate synthase (GS/GOGAT) cycle [33]. Glutamine synthetase (GS; EC 6.3.1.2) catalyzes the ATP-dependent synthesis of Gln from ammonium and Glu. There two known GS isoforms: cytosolic (GS1) and plastid (GS2) ones [17]. Cytosolic isoforms are encoded by a small family of genes and are mainly present in the vascular elements of various organs. They are involved in ammonium recycling at certain developmental stages, such as leaf senescence, and in the synthesis of Gln for transport [34]. In most plants, the plastid isoform of GS is encoded by a single gene and is mostly present in leaf mesophyll, where it participates in ammonium assimilation by nitrate reduction and in reassimilation of photorespiratory ammonium [35]. The amount of ammonium produced by photorespiration in the leaves of C3 plants was estimated to be 5–10 times greater than that reduced from nitrates [36]. As far as is known, gymnosperms, unlike angiosperms, have only the cytosolic isoform of GS [37].

The resulting Gln then acts with 2-oxoglutarate to form two molecules of Glu, the reaction is catalyzed by glutamine: 2-oxoglutarate aminotransferase (i.e., GOGAT, glutamate synthase). This enzyme is present in plants in two forms that differ in electron donor: one uses reduced ferredoxin (Fd) (EC 1.4.7.1), the other uses NADH (EC 1.4.1.14) [38]. Fd-GOGAT is normally localized in chloroplasts and, together with GS2, takes part in reassimilation of photorespiratory ammonium, whereas NADH-GOGAT is mainly localized in non-photosynthetic cells, where, together with GS1, it assimilates ammonium that was absorbed or formed by nitrate reduction or by symbiotic fixation of N_2_ [39]. In most plants, GS1 is encoded by a small gene family of 2–5 members. The gene families of GS2, Fd-GOGAT and NADH-GOGAT consist of as little as 1–2 genes each [40].

In addition to the GS/GOGAT cycle, other enzymes are also involved in the assimilation of ammonium. Unlike GS and GOGAT that catalyze irreversible reactions, glutamate dehydrogenase (GDH) catalyzes reversible amination/deamination, which can lead to either synthesis or catabolism of Glu. GDH has two known main forms: NAD(H)-dependent GDH (EC 1.4.1.2) localized in mitochondria and NADP(H)-dependent GDH (EC 1.4.1.4) localized in chloroplasts [41].

Once nitrogen has been absorbed and assimilated into Gln/Glu, it is transported for storage and utilization, mainly in the forms of Gln, Asn, Glu and Asp, which make up 70% of the free amino acids in plants [42]. Gln and Asn are considered to be the preferred forms for transport because they contain two atoms of N per molecule. Cytosolic asparagine synthetase (AS) [EC6.3.5.4] catalyzes the ATP-dependent transfer of the Gln amide group to the Asp molecule to produce Glu and Asn [43]. Aspartate aminotransferase (AspAT) [EC 2.6.1.1] catalyzes a reversible reaction producing Asp and 2-oxoglutarate from Glu and oxaloacetate, and vice versa [44].

### 3.4. Remobilization of Nitrogen

The ability of plants to effectively remobilize N to ripening fruits or grains is crucial for the overall NUE, especially in cereals, since most N contained in rice or wheat grains (about 80%) comes from leaves [45]. Chloroplast proteins are known to account for about 80% of N stored in leaf tissues, with ribulose-1,5-bisphosphate carboxylase/oxygenase (Rubisco) (a carbon binding enzyme) accounting for up to 50% of N stored in C3 plants and for about 20% of that stored in C4 plants [46]. Thus, remobilization of N formed in senescing leaves (as a result of breakdown of photosynthetic plastid proteins) is a key factor for efficient utilization of N, yet here a dilemma arises [17]. Delayed leaf senescing increases the yield and the carbon content of grains while reducing their protein content. On the other hand, increased N remobilization makes it possible to more fully use N from vegetative parts for grain filling.

The GS1 enzyme has an important role in N remobilization. However, its isoforms differ in localization in plant tissues, regulation and kinetic properties, and hence, not all of them are equally involved in N remobilization [17]. Seeds are the main consumers of remobilized N, which comes to them via the phloem. An essential role in the process belongs to amino acid transporters, namely amino acid permeases (AAPs) [47].

### 3.5. Regulation of Nitrogen Metabolism

The regulation of N metabolism in plants is a rather complex system. In recent years, researchers identified a large number of transcription factors (TFs) that regulate the expression of genes involved in N uptake, transport and assimilation. Over 40 TFs from different families were identified for genes involved in transport, reduction and assimilation of nitrates alone. They have different functions and have been organized into four TF layers in the gene regulatory network that controls responses to NO_3_^−^ [48]. A broader network of nitrogen-associated metabolism comprises 1660 interactions between 431 genes, 345 TFs and 98 promoters [49]. Since N metabolism interacts with a wide variety of different cellular processes, this network also includes genes that regulate carbon metabolism and hormone responses. For example, Dof TF was described as regulators of the C-N balance [50]. In addition, apart from being an essential plant nutrient, nitrate also acts as an important signaling molecule in numerous developmental processes [51]. Ammonium and Gln are also considered as signaling molecules, but information about their signaling pathways is still very limited [52].

The expression of TFs and regulatory enzymes is an attractive means for improving the NUE, as these factors tend to regulate several downstream factors. For example, Arabidopsis TGA1 is a high-level regulator that directly regulates 40% (508/1458) of the N-responsive genes in roots, including both N-metabolic genes and their TF regulators [53]. However, few crops have been studied as well as model plants. A deeper knowledge of N metabolism and its regulation in plants is essential not only for improving the NUE for sustainable agriculture, but also for a better understanding of one of the basic processes in plant physiology.

## 4. Genetic Engineering of Nitrogen Metabolism

Conventional breeding mostly focuses on plant genotypes while ignoring the causes of their high NUEs, whereas molecular approaches focus on studying mechanisms and various factors without considering physiological factors that directly influence the plant productivity [54]. The use of transgenic plants makes it possible to evaluate the NUE improvement at different levels. To improve the NUE using biotechnological methods, it was first proposed to compare the general NUE of control and transgenic plants at both limiting and non-limiting N levels, then assess the physiological mechanism involved using the NUpE or NUtE and, finally, conduct confirmatory tests under field conditions [19]. The development of this research area in genetic engineering once started with exploratory studies in a diverse range of crops. In the last decade, however, the research became more practical plane and concentrated almost exclusively on cereals: rice, wheat and maize (Table 1, Table 2, Table 3 and Table 4).

These crops account for 42.5% of the world’s food calorie supply and 37% of protein supply [158]. However, their NUEs are low and their cultivation requires lots of N fertilizers.

### 4.1. Nitrate Transporters

Almost all transformation studies with nitrate transporter genes were performed in rice. This crop is traditionally grown under flooded anaerobic soil conditions, with NH_4_^+^ being the primary source of N. Owing to its nitrification in the rhizosphere, however, 25–40% of total N can be absorbed as NO_3_^−^, mainly through the HATS system [159]. In rice, according to current knowledge, there are 94 transporters in the NRT1/NPF family, 5 in NRT2, 5 in CLC and 9 in SLAC1/SLAH families [160]. In one of the first studies on plant transformation with nitrate transporter genes, an *OsNRT2.1* gene, which encodes the main high-affinity nitrate transporter, was overexpressed under constitutive the CaMV 35S promoter (p35S). This increased the biomass of rice seedlings under hydroponic conditions, but did not enhance the NO_3_^−^ uptake by plants [55]. Later, this gene was transferred to rice under a ubiquitin- (pUbi) or NO_3_^−^-inducible promoter of an *OsNAR2.1* gene encoding a nitrate transporter partner protein [62]. RNAi knockdown of the *OsNAR2.1* gene showed that it regulates the activity of both HATS and LATS [56]. The *OsNRT2.1* gene expression under each of the promoters significantly increased biomass and yield in the field. The agricultural NUE declined significantly in plants with the pUbi but increased significantly in plants with the *NAR2.1* promoter [62]. Considering its efficiency, the *OsNAR2.1* gene itself was overexpressed in rice plants under a native promoter, and this improved NO_3_^−^ uptake, yield and NUE [63]. Furthermore, a study was conducted to compare rice plants modified with either one or both of the *OsNAR2.1* and *OsNRT2.3a* genes under the p35S [69]. In the field, the yield in double transformants increased more than in plants with only the *OsNAR2.1* gene; in plants with only the *OsNRT2.3a* gene, the yield did not differ from control. The effect of co-overexpression of the *OsNAR2.1* and *OsNRT2.3a* genes was similar in different rice cultivars, and the authors supposed that this approach can also be successful with other crops.

Several studies investigated spliced isoforms of nitrate transporter proteins. As found in [60], overexpression of the *OsNRT2.3b* isoform improved the grain yield and NUE, while that of *OsNRT2.3a* had no effect on these parameters. The OsNRT2.3b splice form acts to switch nitrate transport activity on or off by a pH-sensing mechanism, which improves pH homeostasis and adaptation to changes in the availability of N forms in the environment. A comparison of overexpression of either *OsNRT1.1a* or *OsNRT1.1b* showed that both splice forms caused an increase in biomass under hydroponic conditions [61]. Yet, based on assessments of N accumulation in transgenic plants grown at different concentrations of NO_3_^−^ and NH_4_^+^, the authors concluded that OsNRT1.1b would improve growth even at low N levels, unlike OsNRT1.1a. A study by Huang et al. [65] demonstrated that *OsNPF7.7* had two splicing variants (*OsNPF7.7-1* and *OsNPF7.7-2*) that are differently expressed at the reproductive and vegetative stages, are localized in plasma or in vacuolar membranes and facilitate the influx and concentration of, respectively, NO_3_^−^ and NH_4_^+^ in root. However, overexpression of each variant improved NUtE and could enhance rice grain yield by increasing the tiller number.

The use of natural variations of nitrate transporters offers interesting prospects for improving the NUE. According to Hu et al. [58], NRT1.1B (OsNPF6.5) diverges between two main rice subspecies, *indica* and *japonica*, and this divergence could have probably occurred during rice domestication. The *NRT1.1B-indica* variation was associated with higher NO_3_^−^ absorption activity of the subspecies, compared to *japonica*, and a transfer of this allele to the *japonica* subspecies improved its grain yield and NUE in the field. A study by Tang et al. [66] found a rare natural allele *OsNPF6.1^HapB^* in wild rice. The allele is trans-activated by the TF OsNAC42 and has been lost in 90.3% of rice varieties due to the overuse of N fertilizers. Its overexpression significantly increased rice yield, while its knockout with CRISPR/Cas9 significantly decreased yield, which suggests its role in the activation of NO_3_^−^ uptake.

Arbuscular mycorrhizal (AM) fungi are beneficial symbionts of many plants, including rice, providing the host with nutrients. As shown in Wang et al. [67], up to 42% of overall N absorbed by rice plants inoculated with AM fungus and grown on NO_3_^−^ in pot culture can come through mycorrhiza. Rice with overexpression of the *OsNPF4.5* gene under the pUbi increased the biomass in hydroponic solution with NO_3_^−^, but not with NH_4_^+^ [67]. A knockout of *OsNPF4.5* using CRISPR/Cas9 significantly reduced the shoot biomass of rice in pots with AM fungus compared to WT plants. An experiment with ^15^NO_3_^−^ showed the reduction of symbiotic N uptake in knockout mutants by 45% and the authors suggested that NPF4.5 plays a key role in mycorrhizal NO_3_^−^ acquisition.

Studies were also conducted with peptide transporter genes. Overexpression of the *OsPTR9* gene (*OsNPF8.20*) in rice resulted in increased biomass and crop yield in the field, while RNAi suppression of the gene had the opposite effect [57]. The transporter OsPTR6 (OsNPF7.3) transports the di/tripeptides Gly-His and Gly-His-Gly, and its overexpression in rice increased the biomass under hydroponic conditions at different N concentrations [30]. However, the NUE did not differ from control and even decreased significantly at excessive levels of NH_4_^+^ as the sole N source. In the field, such plants increased not only the biomass and yield but also the NUE [64]. There are also not many studies dealing with the effects of nitrate transporter gene overexpression in other cultivated crops. Among other things, they reported increased biomass and fruit weight in tomato plants with the *LeNRT2.3* gene [59] and increased protein content in young tubers of potato plants with the *StNPF1.11* gene [68].

Quite rare studies use manipulations for N (especially NO_3_^−^) remobilization rather than its uptake or metabolism. A new strategy for specific enhancement of nitrate remobilization was developed in Chen et al. [70]. For this purpose, the nitrate transporter NRT1.7 (NPF2.13)—which is expressed in the vein phloem of senescent leaves and is responsible for remobilization of accumulated NO_3_^−^—was substituted with a hyperactive nitrate transporter NC4N, in which four transmembrane domains of the low-affinity NO_3_^−^ transporter NRT1.2 (NPF4.6) of *Arabidopsis* had been replaced by the corresponding CHL1 region. The chimeric transporter NC4N showed hyperactive low-affinity NO_3_^−^ uptake, as well as significant high-affinity uptake activity, although lower than that of CHL1. The yield of rice with the NC4N transporter in the field increased by 8–11% due to increased panicle number per clump and grain number per clump. This shows that enhanced source-to-sink remobilization of NO_3_^−^ is a new strategy for improving the NUE in crops [70].

### 4.2. Ammonium Transporters

Uptake of NH_4_^+^, the other important form of N, is mediated by ammonium transporters (AMTs). The relevant studies were mostly conducted in rice, for which NH_4_^+^ is the preferred form of N. *OsAMT1.1* is the most active and/or most N-responsive gene of high-affinity NH_4_^+^ membrane transporter, but its overexpression under the pUbi in rice hydroponically grown for 21 days at low or high NH_4_^+^ levels did not change or significantly reduced the biomass compared to control [71]. This could have happened because roots at the early stage of growth were unable to assimilate large amounts of absorbed NH_4_^+^, which had a toxic effect; with a longer cultivation, the reduced biomass of transgenic plants began to restore [72]. The findings were supported by [73], where rice plants overexpressing the *OsAMT1;1* gene were grown in hydroponics at low (30 µM), optimal (300 µM) and high (3000 µM) NH_4_^+^ levels, and the yield increased by >30% and 20% under low and optimum NH_4_^+^ levels, respectively. At a high NH_4_^+^ level, poor growth and over 95% empty grains were observed both in transgenic and WT plants due to NH_4_^+^ toxicity. In addition to NH_4_^+^ toxicity, the plant growth disorders could have been caused by a disturbance of the C-N metabolic balance due to an overexpression of NH_4_^+^ transporters. Rice plants with overexpression of high affinity NH_4_^+^ transporter AMT1-3 showed a significant reduction in growth and yield, which was associated with changes in the C/N balance, soluble proteins and carbohydrates, C and N metabolites [74]. More encouraging results were obtained in maize, where overexpression of the *ZmAMT1;1a* gene notably enhanced the high-affinity NH_4_^+^ uptake capacity in roots under low NH_4_^+^ supply [75]. This increased the biomass (due to the growth of roots rather than shoots), but there were no changes at high NH_4_^+^ levels. In general, attempts to improve plant growth through the use of NH_4_^+^ transporters were unsuccessful and were not brought to field trials.

Overexpression of urea transporters in crops was not reported, but knockout of a urea transporter DUR3 in retrotransposon Tos17 insertion rice lines reduces yield in both hydroponic and field due to decreased grain filling [161]. Because shoot biomass production was not reduced, these results indicate that DUR3 is involved in N distribution to panicles during heading.

### 4.3. Nitrate and Nitrite Reductases

Expression of nitrate reductase genes was mostly used to reduce nitrate content rather than to increase the NUE. NO_3_^−^ is reduced to NO_2_^−^, which can oxidize hemoglobin in blood; it can also react with amines to form carcinogenic nitrosamines. A transfer of a tobacco *Nia* gene under the p35S led to a significant decrease in leaf nitrate content in four lettuce varieties [76]. In another study, however, a transfer of the same gene to another lettuce variety did not have similar effect, possibly due to transgene-specific silencing [80]. Under greenhouse conditions, potato plants with a chimeric tobacco NR gene demonstrated a decrease in the NO_3_^−^ content by 90–98% in tubers, depending on the N content in nutrient solution [77]. The effect was confirmed in the field: the tuber nitrate content decreased by about 95% [78]. A more detailed study of the effects of the chimeric *NR* gene expression in potato in the greenhouse showed not only a decrease in nitrate content, but also an increase in biomass accumulation, both total and in tubers [79]. The authors concluded that a higher rate of NO_3_^−^ reduction improves allocation of N resources for photosynthesis and carbon metabolism.

Overexpression of the tobacco nitrate reductase gene in a monocotyledonous plant, wheat, was studied by Zhao et al. [81]. Under greenhouse conditions, the transgenic plants showed a significant increase in grain protein content and 1000-grain weight (TGW), probably due to accelerated NO_3_^−^ assimilation, which facilitated N flow into seeds and increased the photosynthetic capacity. The allelic variability of the nitrate reductase *OsPG2* gene was shown to be the main cause of the differences between *indica* and *japonica* in the ability to assimilate NO_3_^−^ and in the NUE [84]. The higher activity of *indica* NR can explained by the presence of arginine in the NAD(P) binding domain; in *japonica*, it is substituted with tryptophan. Overexpression of the *indica OsNR2* allele in *japonica* rice significantly increased—while its RNAi suppression significantly decreased—the yield in the field due to change in the effective tiller number. The NUE was further enhanced by the promotive feed-forward amplifying functional relationship between the *indica OsNRT1.1B* (nitrate uptake transporter) and *OsNR2* alleles, demonstrating reciprocal regulation of NO_3_^−^ uptake and assimilation functions [84].

Burley tobacco market type, which is used for cigarette production, displays a chlorophyll-deficient phenotype that requires much higher levels of N fertilizers than other tobacco types to achieve comparable yields [83]. Hence, its leaves accumulate higher levels of free NO_3_^−^, which are associated with the formation of tobacco-specific nitrosamines (TSNAs), some of which have high carcinogenic activity [162]. To reduce the NO_3_^−^ content, genes encoding nitrogen metabolism enzymes (two variants of NR, GS1 and GOGAT) and the ICDH enzyme responsible for the carbon skeleton of 2-oxoglutarate necessary for Glu synthesis were transferred to tobacco, all under the p35S [83]. Of all the constructs, only the expression of the tobacco *Nia2* gene with one amino acid substitution dropped the content of free NO_3_^−^ to about 4% of that observed in WT plants, under field conditions. As a result, the content of total TSNAs in cured leaves and cigarette smoke decreased about four times, without altering the alkaloid profile. Later, the successful construct was transferred into a superior, commercial-grade genotype, and the transgenic plants demonstrated early flowering and a significant reduction in biomass under field and controlled conditions [85]. To mitigate these undesirable traits, the transgenic plants were crossed with two late-flowering cultivars and the resulting F1 hybrids restored the biomass to WT level while preserving the trait of low NO_3_^−^ and TSNA levels.

There are much fewer studies with NiR genes. The Arabidopsis NiR gene under the control of a constitutive CERV promoter has been overexpressed in tobacco to reduce the level of residual nitrite in leaves, which can act as precursor to the formation of TSNAs [82]. When grown in solution containing 10 mM NO_3_^−^, the transgenic plants had a stay-green phenotype and an insignificantly decreased leaf content of nitrite and nitrate. In plants grown at 1 mM NO_3_^−^, nitrite and nitrate levels did not change in older source leaves, whereas nitrate significantly decreased in younger sink leaves of three out of four transgenic lines, and nitrite in one of the four lines.

### 4.4. Glutamine Synthetase

The end products of the GS/GOGAT cycle, Gln and Glu are N donors for the biosynthesis of basic N compounds in plants, including other amino acids, nucleic acid bases, polyamines and chlorophylls [163]. Thus, these enzymes play a key role in N metabolism and are most often used to improve NUE. Their use dates back to 1989, when the alfalfa GS1 gene was transferred to tobacco [164]. Later, the GS genes (mainly the cytosolic form of GS1) were transferred into various plants, but the relationship between the enzyme activity and productivity was ambiguous.

Increased grain weight and grain nitrogen content in wheat, as well as increased biomass and soluble protein content in *Lotus japonicus*, were achieved through overexpression of GS1 genes under a light-induced rbcS promoter [88] or the constitutive p35S [91], respectively. Overexpression of maize *Gln1-3* gene driven by a constitutive CsVMV promoter increased the maize yield in the greenhouse by 30% due to a greater number of kernels [93]. The dry shoot mass did not change and the authors suggested that the effect of the *Gln1-3* gene is specific to grain production. Barley lines overexpressing native *HvGS1.1* demonstrated a significantly higher yield when grown in the greenhouse under low (0.2 g N/L soil) or high N (0.6 g N/L soil) supply. Under excessive N supply (1.0 g N/L soil), however, grain yield did not differ from control [100]. Transgenic plants did not differ from control in terms of vegetation biomass and had a significantly higher NUtE at all N concentrations.

On the other hand, overexpression of a soybean GS15 gene under the p35S in *Lotus* *corniculatus* did not affect biomass accumulation [86], and caused a significant biomass reduction under a root-specific rolD promoter in *Lotus japonicus* [87]. The same gene fused with the constitutive promoter (p35S), a putative nodule-specific promoter (LBC3) or a putative root-specific promoter (rolD) had no consistent positive effect on biomass accumulation in hydroponically grown pea at different concentrations of NO_3_^−^ [90] or NH_4_^+^ [92]. This soybean gene was probably not suitable for improving N assimilation in other plants of the *Fabaceae* family. It is also possible that the effect on the pea yield could be more favorable, but this parameter was not evaluated. The expression of the *Gln1* gene under control of a pUbi in rice did not change the NUtE of greenhouse grown transgenic plants and did not improve the NUE under N deficiency [95].

The above studies were conducted under controlled conditions, mainly in hydroponics, and the effect might have been different in the field. Overexpression of *GS1* possibly caused a metabolic imbalance; hence, there is a need for better overexpression strategies [165]. For example, hydroponically grown rice plants overexpressing *GS1;1* or *GS1;2* genes under the constitutive p35S showed a significant reduction in root and shoot biomasses and seed yield at all four studied concentrations of N. This could be due to a C-N imbalance and impaired stem to leave transport of N [96]. The metabolic interactions between N and C were shown in [101] in alfalfa plants overexpressing genes of key enzymes involved in sucrose biosynthesis in leaves (sucrose phosphate synthase) or in NH_4_^+^ assimilation in root nodules (GS) under the p35S. Both groups of transgenic plants showed a significant increase in biomass and its better quality due to decreased content of fiber and insoluble lignin and increased content of crude protein and the relative feed value. The authors supposed that the two groups of plants have a common signaling mechanism, which regulates the expression of key genes targeted at two different metabolic pathways, of nitrogen and of carbon [101].

The influence of the environment on the NUE in transgenic plants has been demonstrated many times. Hydroponically grown transgenic rice plants with different GS genes (isoforms *GS1;1* and *GS1;2* from rice and *glnA* from *Escherichia coli*) under the p35S showed increased amino acid content, whereas in the field, the latter decreased significantly in all three transgenic lines, as did the grain yield, although the shoot biomass did not change [94]. In Urriola et al. [97], the biomass and yield of sorghum plants overexpressing a cytosolic GS (*Gln1*) gene under control of a pUbi differed significantly between greenhouse experiments in winter and spring. The environmental effect on NUE was later demonstrated in extensive field trials (over 5 years in ten different locations) on maize overexpressing two copies of a *Gln1-3* (GS1) gene in the mesophyll cells (CsVMV promoter) and in the bundle sheet (RbcS promoter) [103]. The yield of transgenic plants increased by an average of 3.8%, but this increase depended on both environmental conditions and the transgenic line used.

Unlike multiple studies on transfer of cytosolic GS genes, there are very few studies on plant transformation with chloroplast isoform of GS. A transfer of the *GS2* gene cDNA of Chinese cabbage (*Brassica campestris*) to Chinese cabbage increased the content of free amino acids considerably [98]. In another study, wheat plants were transformed with a *TaGS2-2Ab* gene, which encoded GS with a higher activity than that in other wheat haplotypes. In field experiments, overexpression of the gene driven by its own promoter significantly improved grain yield under both low and high N conditions and also had a prolonged flag leaf functional duration [99].

In addition to the overexpression of GS genes of various origins, their function was studied using genome editing technology. Michno et al. [166] was probably the first study with N metabolism genes. It showed a successful mutagenesis of a target *GS1* gene in soybean hairy-root tissue using the CRISPR/Cas9 system. The CRISPR/Cas9 system was used to obtain wheat mutants with three knocked out homologous *TaGS1.1* genes (*GS1.1-6A*, *-6B* and *-6D*) [102]. Field-grown mutant plants were found to be imbalanced by N metabolites, which resulted in yield reduction due to reduced number of spikes and grains under low and high N conditions. The study showed that TaGS1.1 is important for N assimilation and remobilization and necessary for wheat adaptation to N-limiting conditions and for spike development.

### 4.5. Glutamate Synthase

Unlike GS genes, GOGAT genes have been rarely used in plant genetic engineering. A transfer of the NADH-GOGAT gene from *japonica* rice to *indica* rice, which contains less NADH-GOGAT in its sink organs, helped increase grain weight in some lines, indicating that NADH-GOGAT is, indeed, a key enzyme in N utilization and grain filling in rice [104]. The great importance of GOGAT genes for normal plant growth and development was shown in Bi et al. [167], where homozygous rice mutants of CRISPR/Cas9 with the knocked-out *ES7* gene encoding a ferredoxin-dependent glutamate synthase (OsFd-GOGAT) were characterized by severe growth retardation and died at the seedling stage.

In recent studies, the GOGAT gene was transferred together with other genes involved in nitrogen metabolism. In rice plants, the expression of the *OsAMT1;2* and *OsGOGAT1* genes was enhanced by isolating T-DNA activation tagging mutants with inserted enhancer elements, following which the double activation mutants were generated by crossing [105]. The biomass and TGW in field-grown single mutants were significantly lower than in control, whereas those in double mutants did not differ from control. In all mutants, grain protein content and amino acid content were significantly higher than in control. When grown in pots under outdoor N limitation growth conditions, single mutants were similar to control, while double mutants showed improved growth and increased yield and grain protein content. Thus, double activation of *OsAMT1;2* and *OsGOGAT1* can improve the absorption of NH_4_^+^ and N remobilization by seeds [105]. While the overexpression of AMT genes was generally unsuccessful, this strategy will avoid toxic levels of NH_4_^+^ because its uptake is accompanied by increased N assimilation.

Maize plants containing one (NADH-GOGAT), two (NADH-GOGAT + NAD-IDH), three (NADH-GOGAT + NAD-IDH + GDH) or four (NADH-GOGAT + NAD-IDH + GDH + two GS1.3 copies) genes were also obtained [106]. In addition to N metabolism genes, this research also used a gene of isocitrate dehydrogenase (IDH). Two forms of the enzyme, NAD-dependent (EC 1.1.1.41) and NADP-dependent (EC 1.1. 1.42), catalyze the synthesis of 2-oxoglutarate in the TCA cycle that serves as a source of C for the assimilation of NH_4_^+^, in mitochondria or chloroplasts, respectively [168]. In transgenic lines, there was a decrease in shoot biomass and, to a lesser extent, yield, although the TGW did not change. The authors believe that synthesis of 2-oxoglutarate is a key junction point of the carbohydrate and amino acid metabolic pathways, and that its accumulation upsets the balance of the primary metabolism of C and N, which affects the yield of maize.

### 4.6. Glutamate Dehydrogenase

In higher plants, glutamate dehydrogenase (GDH) has a lower affinity to NH_4_^+^ than GS, and hence, they mainly assimilate N in the form of NH_4_^+^ via the GS/GOGAT pathway. In bacteria and fungi, NADP(H)-GDH is more affine to NH_4_^+^; hence, their enzyme more efficiently uses NH_4_^+^ [132]. These considerations were the basis for transferring the GDH genes from various microorganisms, mainly fungi, to plants.

As demonstrated in Lightfoot et al. [107], transgenic maize plants with the *gdhA* gene from *E. coli* had higher height and yield in the field at all three N application rates. Rice plants modified with a GDH gene of *Aspergillus niger* fungus demonstrated direct enzyme assimilation of NH_4_^+^ absorbed from roots, which led to a significant increase in the yield under field conditions, although the TGW did not change [108]. Increased assimilation of NH_4_^+^ was also confirmed by Zhou et al. [169], where the affinity of the fungal enzyme MgGDH from *Magnaporthe grisea* to NH_4_^+^ was dramatically higher than that of the rice OsGDH. As a result, the transgenic rice plants accumulated much less NH_4_^+^ under dehydration stress conditions, i.e., MgGDH expression prevented toxic accumulation of NH_4_^+^. In some cases, the transfer of fungal genes had no significant effect on yield. Du et al. [110] noted a slight decrease in height and biomass in transgenic rice plants modified with the *SsGDH* gene from *Sclerotinia sclerotiorum* fungus under a series of low-N condition treatment (50, 500 and 5000 µM). Rice plants with a *PcGDH* gene from *Pleurotus cystidiosus* fungus showed no change in the yield in the field [111]. However, these plants, had a considerably higher TGW as well as higher levels of prolamine and glutelin, the cardinal rice seed storage proteins, which improved the seed nutritional value.

A number of studies have demonstrated the advantages of transgenic versus control plants under N deficiency conditions. A transfer of a *gdhA* gene from *Aspergillus nidulans* to potato led to an increase in both total dry biomass and tuber biomass in the greenhouse [109]. Interestingly, with normal N availability, only one line had an increase in biomass, whereas at lower N levels, four other lines did. The transgenic potato plants had a higher NUE than WT, the difference being greater under N deficiency. A transfer of a GDH gene from *Cylindrocarpon ehrenbergii* fungus to rice helped increase the yield in the field by 36–68% upon fertilizing with 37.5 kg N/ha. However, the yield did not change or even decreased significantly upon fertilizing with 112.5 kg N/ha [112]. Similar results were obtained with the GDH gene of *Eurotium cheralieri* fungus: remarkable increases in yield and the TGW were observed at low N fertilization levels (0 and 37.5 kg N/ha), with no changes at a high level (112.5 kg N/ha) [113]. In contrast to these findings, a transfer of the GDH gene from the fungus *Trichurus* to rice led to a considerable increase in yield and the TGW at all studied N fertilization levels (0, 37.5, 112.5 and 187.5 kg N/ha), although the gain decreased with increasing N availability [114]. Plant height never differed from control, but the content of glutelin and prolamine increased at all fertilization levels.

These results strongly suggest that heterologous expression of a bacterial or fungal GDH in higher plants can improve their growth and grain yield due to increased assimilation of NH_4_^+^ and, with some genes, at low N levels as well.

### 4.7. Asparagine Synthetase

Asparagine is one of the most important amino acids for long-distance transport of N in plants; it has a higher N:C ratio and a better stability, solubility and mobility than other amino acids [119]. In plants, Asn not only delivers N from root to shoot, but also re-locates it from senescence organs to growing leaves and developing seeds. There are two forms of asparagine synthetase (AS): AS-A, which uses NH_4_^+^ as the source of N, and AS-B, which uses Gln or NH_4_^+^ [170]. As shown in one study [89] in *Lotus japonicus* plants with an antisense cytosolic GS gene under a soybean nodule-specific LBC3 promoter, the reduction in GS activity correlated with an increase in amino acid content of the nodules, primarily due to Asn. This suggests that when the activity of GS declined, it was substituted by AS, which is also capable of assimilating NH_4_^+^.

In vascular plants, Asn synthesis is catalyzed by Gln-dependent AS (Gln-AS, EC 6.3.5.4). Most transformation studies used the AS-A gene [EC 6.3.1.1] from *E. coli*, which uses NH_4_^+^ rather than Gln to synthesize Asn, does not depend on light and can offer a more effective NH_4_^+^ assimilation pathway [118]. Under the control of various promoters, the bacterial gene was transferred to various dicotelydonous agricultural species (Table 3). The plants were evaluated under controlled conditions with ambiguous results: *Lotus corniculatus* demonstrated a reduction in the content of free amino acids, growth retardation and premature flowering [115], whereas oilseed rape showed increased amino acid content but delayed growth under limited N availability [116]. In transgenic lettuce plants, however, the biomass increased and the NO_3_^−^ content decreased 2.8 times, a valuable trait for this crop [117]. When transferred to tomato, the gene reduced the biomass of transgenic plants by 40–50% at NO_3_^−^:NH_4_^+^ = 6:0.5 in the nutrient solution. The biomass of transgenic plants did not change at NO_3_^−^:NH_4_^+^ = 3.5:3, whereas the biomass of WT plants decreased by 40–70% [118]. The authors suggested that induction of photorespiration and dark respiration resulted in an increased capacity to (re)assimilate NH_4_^+^.

Only recently have researchers begun to use an AS gene from eukaryotes, namely *OsASN1* from rice. In rice mutants generated by CRISPR/Cas9 system, the plant and tiller number were significantly lower compared with WT [119]. These results suggest that OsASN1 is involved in rice development regulation and is specific to shoot growth. Rice overexpressing *OsASN1* did not differ from WT in morphology or yield in the field, yet its grain quality improved significantly due to a 20% increase in protein content and a 13–27% increase in amino acid content [120].

### 4.8. Alanine Aminotransferase

Although alanine is rarely viewed as the key amino acid in the N metabolism, it can be the main storage amino acid under certain stresses (e.g., hypoxia) and play an important role in N storage [171] (Good and Muench, 1993). Hence, attempts have been made to enhance NUE through overexpression of alanine aminotransferase (AlaAT) [EC 2.6.1.2], an enzyme that catalyzes reversible conversion of glutamate and pyruvate to alanine and 2-oxoglutarate [172]. Nearly all relevant studies used a barley AlaAT gene (*HvAlaAT*) (Table 3). Canola plants with this gene driven by a *Brassica napus* root-specific promoter (*btg26*) demonstrated increased biomass and seed yield both in laboratory and field conditions under low N availability. No differences were observed under high N levels [121]. Thus, equivalent field yields could be achieved with a 40% less N fertilizers applied. The AlaAT gene under a homologous *OsAnt1* promoter was introduced into rice, and the transgenic plants showed an increase in biomass and yield under hydroponic conditions with sufficient N supply [122,123]. Later, transgenic rice plants with *HvAlaAT* under the *OsAnt1* promoter were tested in the field for three growing seasons, in lowland and upland conditions. The yield increased considerably, including under limited N supply, probably due to increased N uptake at the early stages of plant development [125].

In addition to rice, the AlaAT gene was transferred to other monocotyledonous plants. The *HvAlaAT* expression increased the biomass in sugarcane [124] and the vegetative biomass and grain yield in wheat, yet it did not change the phenotype of sorghum [126]. As shown by a recent study [128], a transfer of a gene combination of *OsAnt1::HvAlaAT* to rice, wheat and barley increased biomass and yield. Researchers found significant changes in carbohydrate metabolism, which includes glycolysis and the TCA cycle, and suggested that the increased production of energy resulted in increased N assimilation. In addition to a rice tissue-specific (root epidermis) promoter (*OsAnt1*), *HvAlaAT* was also expressed under other promoters. The use of a constitutive UBI4 promoter from sugarcane [126] did not change the phenotype of wheat under hydroponic conditions, although it increased its field yield. On the other hand, *Brassica napus* plants with the *HvAlaAT* gene under the constitutive p35S were phenotypically similar to WT plants under hydroponic conditions, whereas plants under the root-specific *btg26* promoter showed an increase in total biomass [121].

A recent study by Sisharmini et al. [127], with an AlaAT gene from dicotyledonous plants, investigated the possible functional conservation of the gene across monocotyledons and dicotyledons. The AlaAT gene from cucumber (*CsAlaAT2*) transferred to rice under the same promoter *OsAnt1* increased the biomass by up to 27.4%, the grain yield by up to 27.9%, and the NUE up to 107.4% compared with WT, in the greenhouse. These findings are indicative of conserved functions of the gene in monocotyledons and dicotyledons [127]. Overexpression of AlaAT gene increased NUpE in transgenic rice plants [123]. The NUEs of two sugarcane lines also significantly improved compared to wild-type control [124]. In addition, the transgenic lines differed in terms of their NUpE and NUtE contributions to the NUE, and such genotypes may serve valuable tools for understanding these processes. These studies showed that the NUE improvement can be achieved by altering the metabolic pathways downstream of primary N uptake and metabolism.

### 4.9. Aspartate Aminotransferase

Aspartate aminotransferase (AAT) plays an important role in regulating C and N metabolism in various organisms. While bacteria have only one AAT isoenzyme, plants contain multiple AAT isoenzymes located in different subcellular compartments, such as mitochondria, chloroplasts/plastids and cytosol [173]. This enzyme has many similarities with another aminotransferase (AlaAT) but overexpression of AAT did not improve the NUE, in contrast to that of AlaAT. Zhou et al. [129] overexpressed in rice separately all three *AAT* genes from rice and one *AAT* gene from *E. coli* under the p35S. The transgenic plants showed no significant changes in biomass and seed production compared with WT both under hydroponic and field conditions. Later, transgenic *Brassica napus* lines over-expressing AAT from *Medicago sativa* under the salt-stress inducible *btg-26* promoter were produced and analyzed under both high and low N conditions [44]. Only one transgenic line of the six tested showed a significant increase in both root and shoot biomass. It remained unclear whether these alterations were due to overexpression of AAT, or the result of changes during the generation of transgenic plants.

### 4.10. Amino Acid Transporters

Amino acids are one of predominant forms in which organic N is transported at long distances and redistributed in plants, and their efficient delivery is essential for growth and development of vegetative and reproductive organs. Amino acids are transferred by transmembrane amino acid transporter proteins (AATs), the best-studied of which is amino acid permease (AAP) with broad substrate specificity [174]. Suppression of the *StAAP1* gene in potato using an antisense approach did not cause any phenotypic deviations or yield reduction, although the free amino acid content of potato tubers reduced by 50% [130]. This study showed, for the first time, that a decrease in the expression of one AAP family member leads to a decrease in all amino acids. The effect of *AAP1* gene overexpression was further intensively studied in legumes. In particular, an *VfAAP1* gene from *Vicia faba* under control of a seed-specific promoter LeB4 of the legumes was transferred to *Pisum sativum* and *Vicia narbonensis*. In greenhouse-grown transgenic plants, seed yield and total free amino acid content did not change, whereas the relative content of Asn, Asp, Gln and Glu increased, as did the seed protein content, the individual seed size and the vegetative biomass [131]. The authors inferred that seed protein synthesis is N-limited and that N uptake by seeds limits the rate of seed storage protein synthesis. Later field tests confirmed the increase in N and globulin levels in seeds, but it was accompanied by compensatory changes in sucrose/starch and individual seed weight [132].

Insertion of additional copies of the *PsAAP1* gene into pea under control of an *Arabidopsis* AAP1 promoter did not change the seed weight, although it significantly raised the yield by increasing the number of both pods and seeds per pod, and the seed protein content [135]. The study confirmed that amino acid import into cotyledons limits protein levels in seeds. Cultivation of same plants at low, moderate or high N application regimes showed increased biomass production and seed yield, regardless of nitrogen background, which made it possible to achieve the same yield with half the amount of nitrogen fertilizer [136]. However, the causes of the NUE increase were different: in low N soil, it was achieved due to the improved NUtE, in high N soil—due to the improved NUpE, in moderate N soil—due to both the improved NUpE and NUtE. This demonstrates physiological plasticity in response to changes in N content in the soil.

In addition to yield, grain protein content (GPC) is another important parameter for cereal crops—it determines the nutritional value. As shown in the field tests in rice with overexpression or RNAi suppression of an *OsAAP6* gene under the p35S, this gene acts as a positive regulator of GPC, significantly increasing the levels of glutelins, prolamins, globulins and albumins, without altering other agronomic traits [134]. Later, a group of Chinese researchers published a series of papers on their study of the functions of several other rice AAPs. Genes of AAP3, AAP5, AAP1 and AAP4 were overexpressed under the p35S, suppressed by an RNAi construct with a pUbi, and knocked out using CRISPR/Cas9 technology in a *japonica* rice variety [137,138,141,142]. Overexpression of *OsAAP4* or *OsAAP1* gene contributed to an increase in tillering and grain yield due to increased content of neutral or neutral and acidic amino acids, respectively. Suppression of these genes by RNAi and CRISPR resulted in the opposite phenotype. Fang et al. [142] also noted that OsAAP4 positively regulated shoot bud regrowth, probably by coordinating nitrogen and phytohormone pathways. The *OsAAP3* and *OsAAP5* genes had an opposite function: grain yield increased in lines with blocked expression. Overexpression of the *OsAAP5* gene resulted in accumulation of basic (Lys, Arg) and neutral (Val, Ala) amino acids, while that of the *OsAAP3* gene caused accumulation of amino acids Lys, Arg, His, Asp, Ala, Gln, Gly, Thr and Tyr, leading to the suppression of bud growth and tillering in rice. These studies also showed some divergence between *japonica* and *indica* rice in the promoter sequences of *OsAAP3*, *OsAAP5* and *OsAAP4* genes, which caused differences in expression and, hence, in the number of shoots in these rice subspecies [137,138,142].

In another important cereal crop, wheat, overexpression of a *TaAAP13* gene under an endosperm-specific HMW-GS promoter resulted in a considerable increase in the concentration of grain nitrogen and TGW, but was associated with a significant decrease in the number of grains per spike, yield and biomass [143]. Suppression of gene expression by RNAi did not affect biomass and yield, although it significantly altered the composition and distribution of metabolites in the starchy endosperm. The authors suggested that the yield was limited by N remobilized from the leaves, and that it could be increased by increasing N remobilization.

Similar to the study by Kaur et al. [101], which investigated interactions between N and C metabolism, a study was conducted to identify interactions between N and C transport. For this purpose, they first obtained homozygous pea lines overexpressing a sucrose transporter (*PsSUT1*) or an amino acid transporter (*PsAAP1(3a)*) gebes and then crossed them [144]. The transgenes contributed to transition of young leaves from sink to source state, and the protein content and seed yield in the double transformants were higher than in the single ones. In addition, the changed C:N dynamics in double transformants has made it possible to extend the harvest window for the pea crop from the usual 2–3 days to 3–4 days.

Besides the well-studied AAP subfamily, the large amino acid transporter family (ATF, also called AAAP) includes several other subfamilies that are usually distinguished based on substrate specificity [32]. Among them, members of the LHT subfamily (lysine and histidine transporters) can not only take up amino acids from soil, but can also transport a broad, though distinct, spectrum of amino acids within plants [175]. Wang et al. [139] showed that the OsLHT1 transporter prefers neutral or acidic amino acids, and its knockout in rice using the CRISPR/Cas9 technology inhibited root and shoot growth and reduced yield by 43–68% in the field. The mutant plants also showed decreased NUpE (by 55%) and NUtE (by 72%), and their seeds contained much more protein and amino acids but had a lower germination rate compared with WT [140].

In addition to non-specific amino acid permeases and moderately specific LHTs, specific amino acid transporters were also used by researchers. It is known that the yield and quality of legume seeds are limited by the amount of sulfur contained as Cys and Met amino acids in seeds, and that the main sulfur transporter in the phloem is the amino acid S-methyl-Met (SMM) [176]. Since plant SMM transporters are not known, pea was transformed with a gene of a high-affinity yeast SMM transporter, S-methylmethionine permease 1 (MMP1) under a phloem-specific AAP1 promoter from *Arabidopsis* [133]. Greenhouse-grown transgenic plants significantly increased biomass and seed yield due to a higher seed number per plant, as well as the total content of S, N and protein in seeds, although the relative content of sulfur in seeds did not change.

### 4.11. Transcription Factors

Studies showed that a number of genes regulate N uptake and assimilation, and some of them have been used to improve the NUE in agricultural plants (Table 4). Calcium-dependent protein kinases (CPKs) act as calcium sensors and are involved in various biological processes, including N metabolism. Overexpression of an *OsCPK12* gene facilitated a significant increase in the biomass of rice plants (due to shoot rather than root biomass) under low N hydroponic conditions [145]. Since N tissue content did not change, the authors inferred that OsCPK12 had no effect on N uptake but rather influenced N metabolism under N deficiency, thereby increasing shoot growth. This effect was confirmed in the field, where rice plants with the *ESL4* gene (*OsCPK12*) had significantly increased yields and NUEs at ultra-low and low (but not standard) N application rates [150]. The authors suggest that ESL4 may function upstream of N-metabolism genes.

The NAC superfamily is one of the largest plant-specific TF families. He et al. [147] isolated wheat nitrate-induced TF TaNAC2-5A capable of binding to promoter regions in N transport and N assimilation genes. Wheat plants overexpressing the *TaNAC2-5A* gene markedly increased the yield and grain N content in the field under low-N treatment. Zhao et al. [148] identified a new TF TaNAC-S in wheat, which was associated with leaf senescence. Overexpression of the gene delayed leaf senescence (stay-green phenotype) in wheat in the greenhouse, which indicates its role as a negative regulator of leaf senescence; however, the yield did not change. At the same time, straw and grain N content increased significantly, reflecting the changes in N remobilization. Zhang et al. [152] identified a *nac7* gene in maize, which encodes an NAC-domain TF associated with N metabolism. Maize plants with RNAi suppression of the gene demonstrated a dark green phenotype under hydroponic conditions. Hybrids between transgenic plants and elite inbred lines were tested for two years in in multi-environment field trials. The trials confirmed the stay-green phenotype and the significant increased yields. Transcriptome profiling showed that NAC7 acts as a negative regulator of the stay-green trait in maize, regulating genes involved in protein turnover, photosynthesis and trehalose-6-phosphate pathways [152].

Maize zinc finger protein Dof1 belongs to the Dof TFs family unique to plants and is a key regulator in the coordinated gene expression involved in carbon-skeleton production necessary for amino-acid synthesis [50]. A transfer of the *ZmDof1* gene under the pUbi to rice led to a great increase in plant biomass under N-deficient hydroponic conditions, with no change in biomass under N-sufficient conditions [146]. The growth improvement was achieved through regulation of genes involved in the TCA cycle and due to increased C flow towards N assimilation. The importance of choice of regulatory elements when using TFs was shown in Pena et al. [149], where the *ZmDof1* gene was transferred to wheat and sorghum under a constitutive UBI4 promoter or under a tissue-specific rbcS1. The tissue-specific expression increased biomass, yield and NUE, whereas the constitutive expression had either neutral or negative effect due to downregulation of genes involved in photosynthesis. Overexpression of *Arabidopsis* TF CDF3 (DOF TF family) in tomato plants promoted a notable increase in the biomass of vegetative organs and fruits in both N-poor and -rich conditions [154]. The authors suggested that DOF genes could play similar functions in C/N metabolism in dicot and monocot species.

TFs from the basic leucine zipper (bZIP) family play an important part in plant growth, development and resistance to biotic and abiotic stresses. As shown in Yang et al. [153], they can be involved in N use regulation. Overexpression of the *TabZIP60* gene reduced wheat yield in the field, primarily due to a decrease in spike number, while RNAi suppression of the gene led to a considerable increase in yield. TabZIP60 was shown to bind with a promoter of the *TaNADH-GOGAT-3B* gene and negatively regulate its expression. A fundamental role of rice Nin-Like Protein 1 (OsNLP1) in N use was also demonstrated [155]. Overexpression of the gene significantly increased rice yield and the NUE in the field under different N conditions, whereas its knockout with the CRISPR/Cas9 system worsened these parameters at low and normal N and did not change them at high N levels. The authors showed that OsNLP1 positively regulates the transcription of genes related to uptake and assimilation of NO_3_^−^ and NH_4_^+^. A recent study by Wu et al. [157] showed that another TF from this family, OsNLP4, is a key regulator of the NUE, coordinating a majority of the genes related to N utilization and signaling.

Semi-dwarf Green Revolution varieties of wheat and rice boast of high yields, and their phenotypes are due to mutant alleles, which contribute to the accumulation of growth-repressing DELLA proteins [151]. In addition, DELLA inhibits N assimilation and thereby reduces NUE. Expression of rice growth-regulating factor 4 (GRF4) was modified in wheat and rice using overexpression, RNAi, and CRISPR/Cas9 approaches. It has been shown that interaction of GRF4 with growth inhibiting DELLA provides a joint homeostatic regulation of growth and C and N metabolism [151]. Enhanced expression of GRF4 contributed to a higher NUE and yield, but without affecting the beneficial semi-dwarfism trait. This opens up new opportunities for increasing crop yields and nutrient use efficiency. Later, it was shown that TF GRF4 can regulate N use through TF MYB61, whose alleles differ in two rice subspecies, *indica* and *japonica* [156]. CRISPR/Cas9-generated mutations in *myb61* and *grf4* genes showed that the *indica* allele of MYB61 displays robust transcription, resulting in a higher NUE and grain yield with less N supply compared with the *japonica* allele.

## 5. Nitrogen Metabolism in Forest Trees and Its Modification

### 5.1. Nitrogen Metabolism in Trees

Efforts to improve NUE are mainly focused on crops while nearly ignoring woody plants. This can be explained by the difficulties of work with tree species due to their large physical size, large genome size (especially in coniferous species), long life cycle including long generation time and problems with in vitro cultivation (for genetic transformation and regeneration of transgenic plants). Yet trees supply valuable materials for various industries and contribute to soil conservation and maintenance of water balance; they are also important for the global carbon budget and maintenance of biodiversity. Modern forest plantations, whose share in wood production is constantly increasing, are highly intensive production systems that use special cultivars, efficient management systems, fertilizers and pest and weed control. In fact, they are already not much different from crop fields. These new forests will require sustainable use of fertilizers, with N as one of the most important nutrients, and therefore, trees with an improved NUE will be required to increase the productivity of future plantations [177].

Nitrogen is a limiting factor for tree growth and development, as its availability in forest soils is low. Forest trees developed a number of mechanisms, e.g., mycorrhizae, to absorb and transport various forms of N [178]. In general, there are no differences between herbaceous and woody plants in the ways of assimilating inorganic N from soil [179]. Trees may prefer NO_3_^−^ or NH_4_^+^, depending on their concentration in soil, the tree species, soil temperature and pH [180]. In boreal forests, however, nitrification is limited by factors such as low temperatures, low soil pH and high residual lignin content, and therefore, NH_4_^+^ is the predominant source of inorganic N for trees [178]. Interestingly, poplar was found to have 16 ammonium transporter genes, which is considerably more than in Arabidopsis (6) or rice (10). This may reflect the physiology of perennial trees with mycorrhizal symbiosis as some AMT genes were overexpressed in response to mycorrhizal inoculation [181,182].

GS is the central enzyme in N metabolism. In deciduous trees, the number, localization and functions of GS isoforms are the same as in most angiosperm species. As shown by studies, *Populus* has one isoform of the plastid GS (GS2) and three isoforms of the cytosolic GS (GS1.1, GS1.2 and GS1.3), which are responsible for the NH_4_^+^ reassimilation in photosynthetic cells, N mobilization during its seasonal recycling and in the biosynthesis of Gln for N transport, respectively [177]. Coniferous trees do not have the GS2 isoenzyme, its function is performed by the cytosolic isoform GS1a, while the GS1b isoform is similar to the GS1 of angiosperms [183].

The process of N remobilization and storage is another specific feature of trees. In annual crops, N from the senescing leaves transports to grain at the end of the season, N content in seeds determines the germination and survival of seedlings. Unlike annual plants, trees translocate up to 50–80% of leaf N to the trunk for storage during seasonal dormancy, and therefore, N storage and recycling processes are especially important for the survival of perennial plants [184]. Trees undergo two annual phases of remobilization: in autumn, N is transported from senescing leaves to the trunk to be stored there through winter, and in spring, it is remobilized from the trunk into the developing organs before the roots begin to supply the tree with enough N [17]. As trees age, this internal cycling of N becomes increasingly important for the overall nitrogen budget of the tree.

In autumn, most remobilized N from senescing leaves is used in the synthesis of bark spare proteins (BSPs), which accumulate in protein storage vacuoles of the inner bark parenchyma and xylem ray cells [185]. Manipulations with BSP production can also contribute to increasing the biomass production in trees. Nitrogen is stored not only in the form of proteins but also in the form of amino acids. Arginine, with its four N atoms per molecule, as well as Gln and Asn, each containing two nitrogen atoms per molecule, is of particular importance as a soluble N storage substance [186]. Nitrogen reserves accumulated in autumn are rapidly mobilized to support growth at the beginning of the growing season. As air warms up faster than soil, budburst starts earlier than the roots begin to absorb sufficient N, and therefore, N stored in the trunk is used [187]. In most tree species, Gln or Asn are usually the main amino acids transported. Yet, depending on the species, other forms of N, such as aspartate, arginine or citrulline, can also be transported [187]. For example, citrulline, a non-protein amino acid with three N atoms per molecule, prevailed in the xylem sap of *Alnus* spp., *Betula* spp. and *Juglans* spp. during N remobilization in spring [188].

Unlike in herbaceous plants, the woody tissues of tree trunks are important sinks for carbon and nitrogen assimilated during the plant’s life cycle [189]. As reported in [190], at low nitrogen levels, excessive carbon is redirected to the biosynthesis of aromatic amino acids and lignin, which results in improved NUE and may be of practical importance for biomass production. A better understanding of the N metabolism in trees cannot be achieved without identifying the regulatory networks and key TFs. It is known that the transcriptional network regulating secondary cell wall biosynthesis in trees is more complex than in herbaceous plants [191], and the same should be expected for the regulation of N metabolism. Among other things, it is not clear how the expression of BSP genes is regulated in trees [184].

### 5.2. Transgenic Modification of Nitrogen Metabolism in Trees

Enhanced ability to assimilate N may be of particular importance for forest trees that have to be grown for many years to obtain the biomass. In conifers, the cytosolic form of GS is present in both photosynthetic and non-photosynthetic tissues [37]. The first attempt to improve the NUE in trees was made in the study [192], where they transferred the gene of pine cytosolic GS (*Pinus silvestris* L.) to a hybrid poplar (*Populus tremula* × *P.alba*) under the p35S. After 6 months of growth in a greenhouse, the height of transgenic plants exceeded the control by 21%. In addition, they had a higher content of total protein and chlorophyll and a larger leaf area [193]. The authors’ hypothesis that GS expression in the cytosol of photosynthetic cells could improve the recycling of NH_4_^+^ released in secondary processes was confirmed in Man et al. [194]. Poplar plants were grown at low (0.3 mM) and high (10 mM) NO_3_^−^ levels, and their leaf biomass increased, respectively, by 112% and 26% versus control. Furthermore, transgenic leaves contained 85% less free NH_4_^+^, but more free Gln and total free amino acids. When grown at 10 or 50 mM NO_3_^−^, transgenic plants significantly increased the aboveground biomass, the effect being greater at 50 mM NO_3_^−^ [195]. The NUpE and NUtE also exceeded the control values, especially in young leaves. Field trials confirmed the greenhouse data: three-year-old trees were 41% taller and 36% larger in diameter compared with control [196]. Determination of bark protein content suggested that transgenic trees accumulated more N in the form of bark storage proteins (BSPs) in their stems after the growth season. The amount of lignin in plants with accelerated growth did not change, and they showed improved wood quality for paper production: a minimum decrease in pulp yield (by 1.5–4%) was accompanied by a significant reduction in lignin residues (the Kappa number decreased by 12–21%) [197]. Field trial findings support the hypothesis that growth acceleration in transgenic hybrid poplar trees with the pine cytosolic GS gene is caused not only by primary N assimilation, but also by re-assimilation of NH_4_^+^ released in various metabolic processes.

Later, a Scots pine *GS1* gene under the p35S was transferred to birch (*Betula pubescens*, *B. pendula*) and aspen (*P. tremula*, *P. tremula* × *tremuloides*) [198]. After two years of growth in greenhouse conditions, transgenic plants showed increased height and wood volume versus control: by up to 23 and 41% in aspen, and up to 41 and 74% in birch, respectively [199]. In an outdoor pot experiment with aspen and birch plants fertilized with 0, 0.1 or 10 mM NO_3_^−^, transgenic plants also demonstrated an increased height gain versus control, although nitrogen fertilization decreased this effect [200]. Determination of biomass N content revealed a substantial increase in NUpE, NUtE and NUE in transgenic birch plants at low N, with no differences vs. control at high N [201]. An earlier study of Man et al. [202] found a marked increase in free indoleacetic acid (IAA) levels in the leaves of transgenic poplar with a *GS1* gene and suggested that the increased level of cellular Gln enhanced the growth of transgenic plants via the regulation of auxin biosynthesis. We evaluated in vitro rooting of birch plants and found that transgenic plants with the GS1 gene rooted faster than controls [203]. The IAA concentration in transgenic plants was three times as high as in control and sharply declined after treatment with GS inhibitor phosphinotricin (PPT) (no change in control), indicating that the enzyme contributed to the increase in auxin levels in transgenic plants. Furthermore, the transgenic birch plants were distinguished by increased content of Gln, Glu and Asp, as had already been noted by Man et al. [194] for transgenic poplar grown at low N. All these results confirm that expression of conifer GS gene in transgenic deciduous trees can improve NUE in N-deficient soil. This strategy can be applied to establish forest plantations on poor soils.

Some other genes associated with N metabolism were also transferred to trees. Unlike the studies in cereals [146,149] or tomato [154], overexpression of the *ZmDof1* gene in poplar had no positive results [204], possibly due to different regulatory systems in annual and woody plants. The gene of Dof5 TF of the same TF family that regulates GS1 isoforms in maritime pine was also transferred to hybrid poplar plants [205]. The transgenic plants showed enhanced aboveground and root growth in a growth chamber, and their NO_3_^−^ uptake was significantly higher than in control. However, two-year field trials showed attenuated growth and no changes in the N metabolism of transgenic trees, possibly due to the fact that the N content in the field soil was 12 times lower than in the potting mix [205].

GATA proteins are eukaryotic TFs that can mediate N metabolism; a GATA gene, *PdGATA19*/*PdGNC,* was identified in a fast-growing poplar clone [206]. Poplar plants overexpressing this gene had significantly increased biomass in the greenhouse, in contrast to the decreased biomass in CRISPR/Cas9 mutants. Transcriptome analysis showed the PdGNC involvement in C metabolism, as well as in NO_3_^−^ transport in roots. Overexpression of a cytosolic NADP+-isocitrate dehydrogenase (ICDH) gene from *Pinus pinaster* in a hybrid poplar (*Populus tremula* × *P. alba*) resulted in a significant increase in plant height under greenhouse conditions and changes in the amino acid content in young leaves, which implies their enhanced biosynthesis [207].

Of interest are strategies for manipulating tree-specific bark storage proteins (BSPs) involved in the seasonal N cycling and used to store N during the dormancy period, but their effect on growth has not been thoroughly studied yet. RNAi suppression of the *BSPA* gene in poplar showed that accumulation of BSP plays a part in N remobilization from senescing leaves to bark in autumn and from bark to expanding shoots in spring [208]. The latter process is apparently regulated by auxin, but further research is needed to identify regulatory and transport factors involved in N remobilization in trees.

## 6. Ammonium Toxicity and Resistance to Phosphinothricin

NO_3_^−^ and NH_4_^+^ are the major forms of N taken up by plants from the soil. High concentrations of NH_4_^+^ are toxic for plants, which is not the case with NO_3_^−^ [27]. Moreover, N is assimilated in plants only in the form of NH_4_^+^, and if the rate of its uptake and release in various cellular processes exceeds that of its conversion into amino acids, its accumulation can reach toxic levels, leading to root growth delay and leaf chlorosis [209]. This aspect should be taken into account in the case of overexpression of NH_4_^+^ transporter genes, when plants do not have enough time to assimilate the absorbed NH_4_^+^, as has already been noted by some researchers [71,73]. The exact mechanism of plant growth disorders due to NH_4_^+^ toxicity is still unknown, although several hypotheses have been put forward, including futile transmembrane NH_4_^+^ cycling, deficiencies in inorganic cations and organic acids, impaired hormonal homeostasis, disordered pH regulation and the uncoupling of photophosphorylation [210]. High concentration of NH_4_^+^ is also one of the causes of plant death due to the use of broad-spectrum herbicides based on phosphinotricin (PPT). PPT (DL-homoalanin-4-yl(methyl)phosphinic acid) is an analog of Glu and irreversibly inhibits GS [211]. Apart from contributing to accumulation of NH_4_^+^, PPT disrupts N metabolism downstream of the enzyme, but the relationship between these processes is presumably very complicated and not yet well understood [212]. Nevertheless, overexpression of GS genes seems to be an attractive strategy not only for improving the NUE, but also for increasing the herbicide resistance in plants.

In one of the first studies on the transfer of GS genes to plants, tobacco transformed with an alfalfa GS gene demonstrated an increased resistance to PPT in vitro and to herbicide treatment, as well as a seven-fold decrease in the NH_4_^+^ level compared with control [164]. Transgenic rice with the *GS1;2* gene also demonstrated resistance to the herbicide Basta, unlike plants with *GS1;1* or *glnA* genes from *E. coli*. This presumably indicates that GS1;1 and GS1;2 play different roles in NH_4_^+^ assimilation [94]. Simultaneous overexpression of rice cytosolic (*OsGS1;1*) and chloroplast (*OsGS2*) GS genes in rice increased the plant resistance to spraying with 0.5% Basta solution [213]. In transgenic plants, the NH_4_^+^ level was initially lower than in control (1.2 and 3.1 μg/mL) and less markedly increased after treatment, which means partial detoxification of excessive NH_4_^+^ by overexpression of the GS enzyme. However, the plants did not tolerate treatment with 1% or 2% herbicide solutions, i.e., their resistance was limited.

Similar results were obtained for other N metabolism enzymes that use NH_4_^+^ as a substrate. Basta treatment of the leaves of *Brassica napus* plants with an *E. coli* AS gene caused a substantial increase in Asn content, but it was not sufficient to render resistance to PPT [116]. Meanwhile, lettuce plants with the same gene tolerated increasing doses of NH_4_^+^ and PPT better than controls [117]. Thus, the excess and/or accumulation of NH_4_^+^ caused by GS inhibition could be compensated by AS-A activity. NADP(H)-GDH of microorganisms has a high affinity to NH_4_^+^ and was, therefore, also considered as a means for protecting plants against high NH_4_^+^ concentrations. A series of studies showed that transgenic plants with this gene are slightly more resistant to herbicide treatment [107,110,214] and to high NH_4_^+^ levels under dehydration stress [169]. For example, the *gdhA* gene from *E. coli* increased the tobacco resistance to glufosinate six times, whereas the herbicide-resistance *bar* gene increased it 100 times [215].

There were almost no such studies in trees. It is known that treatment with PPT-containing herbicide sharply increased the NH_4_^+^ content in poplar [216] and aspen [217]. Transgenic poplar with the *GS1* gene was more resistant to PPT in vitro and to herbicide treatment in a growth chamber, but NH_4_^+^ content was not measured [218]. To assess the PPT resistance, eight transgenic lines belonging to two genotypes of downy birch (*Betula pubescens*) transformed with the pine *GS1* gene were treated with Basta herbicide at a dose equivalent to 2.5 and 5 L/ha (half the standard and standard field doses) [219]. Some lines demonstrated enhanced resistance after low-dose treatment. However, the lack of correlation between NH_4_^+^ levels and plant survival suggested that NH_4_^+^ toxicity was not the main cause of birch plant death after the PPT treatment. This inference was confirmed in Lebedev et al. [220], where the resistance of a birch line with the GS1 gene to 2.5 and 5 L/ha Basta was evaluated for two years. One-year-old transgenic birches treated with low dose developed the damage symptoms (visual defects, low chlorophyll levels and dehydration) slightly slower than in control plants, but they still died although later, than the WT plants. However two-year old transgenic plants lost their foliage after such treatment but survived and resumed growth. Despite these different outcomes, low-dose herbicide treatment did not cause any significant increase in the NH_4_^+^ level in plants with GS1 of either age, compared with water treatment. Neither did it change in transgenic birch plants with the bar gene, fully resistant to the herbicide [220].

There is no consensus on the cause of plant death upon PPT treatment, whether it is accumulation of NH_4_^+^, depletion of Gln and Glu or another, recently suggested third cause. As shown by Takano et al. [221], reactive oxygen species are the main driver for rapid cell death after glufosinate treatment, as ammonia accumulation and changes in amino acid levels are probably secondary effects of GS inhibition. On the other hand, the multiple hypotheses regarding the NH_4_^+^ toxicity [209,222] were recently joined by another one. According to Hachiya et al. [210], the primary cause of NH_4_^+^ toxicity is not its high concentration, but rather acidic stress resulting from assimilation of these high amounts by the plastid form of GS. Whatever the exact mechanisms of NH_4_^+^ toxicity or of the PPT effect, the increase in plant PPT resistance achieved through overexpression of GS or other N metabolism genes is absolutely insufficient for the commercial use of such plants.

## 7. Unintended Effects of Transgenic Plants

Plant genetic engineering is aimed at creating plants with new traits rendered by introducing certain genes into them (intended effects). However, unintended effects are also possible in transgenic plants; they can be associated with gene insertion, random mutations during the transformation and in vitro cultivation, or pleiotropic effects of new protein, and they cannot be predicted [223]. It is quite likely that interference with such a fundamental process, as N metabolism can also bring unexpected consequences, but this issue has been poorly studied so far. It is common knowledge that N fertilizers promote the growth of biomass but delay flowering, which can reduce yields in northern regions with a short growing season. One would expect that improving the NUE using transgenic technologies would also delay flowering, but the reality turned out to be not so unambiguous, and quite different scenarios were demonstrated.

Several studies showed the influence of GS genes on plant development. Overexpression of soybean cytosolic GS in *Lotus corniculatus* growing in an NH_4_^+^-rich hydroponic solution, accelerated flowering in plants which is typical for senescent plants [86]. A study with ^15^N showed that the increase in amino acid accumulation was caused not by enhanced assimilation of NH_4_^+^, but by enhanced protein catabolism. Therefore, the authors concluded that it was this process that accelerated the plant development, since premature flower development is usually characteristic of N remobilization at the early stage of plant senescense. Early flowering was also observed in tobacco with overexpression of the GS2 gene [224]; however, a transfer of the soybean GS1 gene to tobacco caused a significant delay in flowering under low and high N conditions [225]. The influence of the environment on the flowering trait was shown in Urriola et al. [97], where transgenic sorghum plants overexpressing the native cytosolic GS gene showed a significant delay in flowering when grown at low N in a greenhouse winter experiment. In spring, however, there was a significant acceleration of flowering at both low and optimal N conditions.

**Figure 2:**
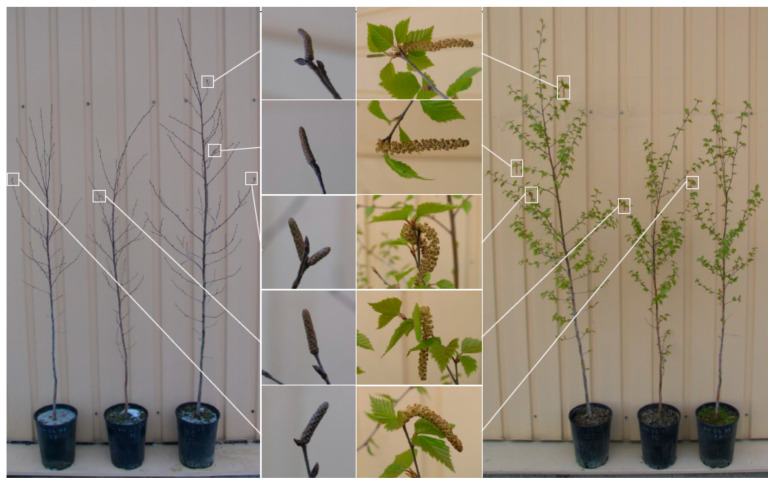
Accelerated flowering of transgenic birch plants with the GS1 gene (line F14 GS 8b at 0, 1 and 10 mM N supply). Left—catkins in November on three-year-old plants; right—flowering in May (beginning of the fourth growing season).

In 2003, Gallardo et al. [189]—based on the early *Lotus* flowering observed by Vincent et al. [86]—suggested that overexpression of GS genes to accelerate flowering would be especially interesting to use in woody plants with a long juvenile period. To our best knowledge, there were no reports of changes in the flowering time of poplar with a pine *GS1* gene, while our studies have identified an early flowering downy birch (*B. pubescens*) line with overexpression of a similar gene. The birch line F14 GS 8b was not only characterized by accelerated growth, but also demonstrated inflorescence formation at the age of three years, whereas normally birch starts blooming at the age of at least 10 years [226]. Flowering was observed in two experiments conducted several years apart and under different conditions: in plants grown on natural soil in a greenhouse for two years and then transferred outdoors [199] and in plants permanently grown outdoors on artificial substrate [227]. In the last experiment, three N treatments were used and early flowering was observed in all of them (Figure 2).

Changes in flowering were also observed in plants overexpressing an AS gene. *L. corniculatus* plants with an *E. coli asnA* gene showed early flowering and growth retardation [115]. The authors attributed these effects to accelerated plant development, as was already noted earlier in *L. corniculatus* plants with a cytosolic GS gene [86]. Acceleration of flowering was also observed in lettuce plants with the same *asnA* gene [117]; *OsASN1* overexpression, however, did not change the flowering time of rice [120], probably because of different effects of overexpression of prokaryotic and eukaryotic AS genes in plants.

The role of nitrate transporters in the regulation of flowering was shown in Wang et al. [228], where *OsNRT1.1A* (*OSNPF6.3*) expression in rice was blocked by T-DNA insertion or RNAi techniques and plants demonstrated significant flowering delay and yield reduction in the field. All these defects were completely rescued by the introduction of *OsNRT1.1A* into the T-DNA insertion mutant background: *OsNRT1.1A* overexpression in WT rice significantly accelerated flowering and increased yield at both low and high N. The gene overexpression in *Arabidopsis* also significantly accelerated flowering. Thus, *OsNRT1.1A* functionally differs from previously described *NRT1.1* genes in plants and it upregulates the expression of N utilization-related genes not only for NO_3_^−^ but also for NH_4_^+^, as well as flowering-related genes [228]. Transgenic *Arabidopsis* plants with a chimeric nitrate transporter gene demonstrated early flowering under N starvation but not under variable or sufficient N supply, which suggested that accelerated NO_3_^−^ remobilization could facilitate flowering under N deficiency [70]. Finally, tobacco plants with overexpression of the NR gene demonstrated an early flowering phenotype, also associated with a considerable biomass reduction, both under controlled conditions and in the field [85]. To prevent these undesirable traits, transgenic plants were crossed with two late-flowering varieties. Plant biomass recovered in both hybrids, but the mechanisms of recovery differed: in one of the hybrids, it was associated with a delay in flowering, whereas the other retained the early flowering trait.

Modification of N metabolism can also impair plant reproductive development. A transformation of *Lotus japonicus* with alfalfa GS1 gene unexpectedly made the transgenic plants sterile due to morphological alterations in pollen grains and in ovules [229]. *GS1* overexpression increased the Gln content in flowers, and hence, increased the Gln/Glu ratio. The authors suggested that a similar metabolic effect had previously caused the sterility of transgenic alfalfa. As highlighted in Schoenbeck et al. [230], NADH-GOGAT is essential to reproductive development: antisense suppression of its expression in alfalfa unexpectedly resulted in pollen sterility in one line due to disturbed metabolism in flower tissues. The study Suarez et al. [229] suggested that sterility was caused by a decrease in high concentrations of Glu and its derivative amino acids, such as proline, necessary for adequate development of flower tissues. Sterility was also found in several maize lines with maximum expression of *E. coli gdhA* gene encoding NADPH-GDH, although the lines with low expression were fertile, but no possible causes were reported [107].

The exact mechanism of the effect of high N doses on delay in flowering is unknown. The study by Wang et al. [228] suggested that high accumulation of NO_3_^−^ and NH_4_^+^ promotes vegetative growth and flowering delay, but as the primary N sources are assimilated, the inhibition of flowering abates. Thus, it is probably the enhanced N assimilation in transgenic plants with improved NUE that accelerates flowering, although more complex mechanisms are not excluded.

The impact of transgenic plants with modified N metabolism on the microbial community of soil has barely been studied so far. How will its activity and biodiversity be affected by a decrease in N content in the soil, either due to increased N uptake by plants or due to decreased N influx with plant residues, which became poor in N due to its accelerated remobilization into the yield? How critical will these changes be for the N cycle on different scales, from a small field to ecosystems of different sizes, and over different periods of time? Being undoubtedly important, such studies are almost never conducted. They are of special value for transgenic trees growing in one place for many years, because even minor changes accumulate with time and can noticeably affect the environment. We assessed the potential impact of such plants on the soil ecosystem in aspen and birch plants with the pine *GS1* gene. They were first grown in pots with natural soil in a greenhouse for two years and then outdoors for another two years. At the end of each growing season, we evaluated activity of 11 soil enzymes involved in C, N, P and S cycles [231]. The differences between the transgenic and control plants were mostly insignificant, except for several lines. The detected differences did not repeat in the subsequent years. The decomposition rate of aspen and birch plants with the *GS1* gene was estimated by measuring the emission of CO_2_ over a period of 12 months [232]. We found an increased decomposition rate in stem tissues of birch due to a low C:N ratio. These cell-level changes can affect the N and C cycles in the ecosystem, but even field trials are not sufficient to estimate the processes. Such global environmental processes involve multiple variables, including random ones. Moreover, the whole process can take place in the context of global climate change whose scenario is also uncertain. Therefore, the development of such ecosystem processes can be assessed only by mathematical modelling. For example, a model scenario of growing transgenic aspen plantations with modified wood composition on the soils of Northern Eurasia for 30 and 60 years did not show possible degradation of forest ecosystems [232]. Chaps in the pools of C and N were in the range of 5–7%, which does not exceed the actual impact of standard silvicultural operations.

By their very nature, many unintended effects can only be detected by chance. For example, extremely low temperatures (down to −13 °C) at the end of October 2014, which occur once in 30 years, caused the death of one transgenic birch line with GS1 regardless of the N supply [227]. We do not know why this happened for sure, but we think that this might have been due to a prolonged growth period and delayed the transition to dormancy. A study of transgenic tree phenology found a delay in budburst in an aspen line with the GS1 gene, compared with other lines and controls [233]. All this indicates the need for a more thorough assessment of transgenic plants, and first of all, the woody ones, before their commercial use.

## 8. Future Prospects

The NUE is a complex polygenic trait. Although multiple and long studies have provided a notable progress in its understanding, there has not been much success in improving the NUE using transgenic technologies. The database of transgenic events approvals for public use [234] currently contains no crops with growth or yield altered through the modification of N metabolism. In addition, fewer studies have evaluated the NUE (Table 1, Table 2, Table 3 and Table 4), which does not clearly link increased yields to an improved NUE. Studies of enzyme gene overexpression, including GS, the key assimilatory enzyme for NH_4_^+^, have had inconsistent results. The inconsistencies may be the consequence of unwanted pleiotropic effects due to the use of constitutive promoters, an imbalance in N metabolite pools, substrate limitations for amino acid synthesis or post-transcriptional and post-translational regulation [11]. Nitrogen metabolism can be modified more efficiently by controlling expression with tissue- or time-specific promoters, as well as TFs capable of simultaneously driving the expression of several genes. Since N uptake and utilization occur independently [18,124], gene pyramiding can provide ample opportunities, and it has already been shown that double transformants improve NUE better than single ones [69,105]. Finally, the development of genomic editing methods also opens up new prospects.

More promising results were obtained with bacterial ASN genes and fungal GDH genes. They are significantly more affine to NH_4_^+^ and are probably insensitive to the plant cell regulatory systems [10]. However, some studies showed that GS overexpression renders tolerance to various abiotic stresses: drought [213,235], salinity [213,236], cold [236], osmotic [213] and heavy metal [237]. Such tolerance is becoming ever more important in the context of ongoing global climate change whose key indicators—rising temperatures, and hence, lower availability of water, as well as rising atmospheric CO_2_—affect the NUE. Water and N are the most important factors in agriculture, but surprisingly little is known about how plants respond to combinations of N and water [238]. The transfer of GS genes rendered plants responsive to increased atmospheric CO_2_. In barley plants overexpressing GS1 and grown under elevated CO_2_ (900 μL/L), the grain protein content was not less than in those grown ambient CO_2_ (400 μL/L), whereas it was significantly decreased in control plants both under low and high N levels [100]. Plants that overexpress GS are more tolerant to various abiotic factors and will probably be better adapted to new conditions of life, and this will compensate for the absence or only slight improvement in NUE. Additionally, of considerable interest is the possibility of accelerating flowering using GS and, probably, other genes. For annual crops, this could help expand production to more northern regions with shorter growing seasons [228]. For perennials, flowering acceleration in breeding is most promising, since a significant (by several years) reduction in the juvenile period will significantly accelerate the breeding cycle, which is very long for woody plants.

Increased atmospheric CO_2_ is known to slow down NO_3_^−^ assimilation in plants [239], while not reducing their use of NH_4_^+^ [240]. Hence, global climate change will increase the importance of NH_4_^+^ in plant nutrition, and there is a need for further research in order to increase its use by crops for higher yield under new conditions. However, one should not forget about toxicity of high NH_4_^+^ levels and take appropriate measures for its prevention. In general, climate change should be taken into account especially for perennial trees, because significant changes in the environmental conditions may occur throughout the life of an individual plant, in contrast to annual species. The environmental conditions at the time of the establishment of a forest plantation can be radically different from those at the end or middle of the rotation.

Many studies aimed at improving NUE were performed under controlled conditions (Table 1, Table 2, Table 3 and Table 4). Their results cannot be fully extrapolated to field conditions where plants have to deal with changes in the environmental conditions and a variety of biotic and abiotic stresses [94,190]. In some cases, e.g., with perennial trees or under unpredictable climate change conditions, even field trials are not sufficient and we have to use mathematical modeling. In this case, the results of field trials (as well as trials under controlled conditions) provide the basis for constructing mathematical models. The use of phenomics methods (high-throughput phenotyping) allow the rapid, non-destructive, objective, large-scale and automated analysis of plants in dynamics and can be of great help here. Quite recent studies have reported the use of high-throughput phenotyping for NUE assessment in conventional cultivars in the greenhouse [241] and in the field [242], but so far, there has been no information on its use in transgenic plants.

Introduction of foreign genes often leads to significant disturbances in N metabolism; therefore, it would be reasonable to use the already available natural genetic variations of the NUE in crop populations. For example, rice has two subspecies: *japonica*, which is more cold tolerant and has a better grain quality, and *indica*, which has much higher yields due to the higher tiller number [138]. Several studies showed the underlying genetic mechanisms behind this difference in the NUE between the two subspecies [58,84,142,156]. Their findings have made it possible to improve the NUE in *japonica* through the use of *indica* gene alleles. It is also known that domestication of rice dramatically reduced the genetic diversity of NH_4_^+^ and NO_3_^−^ transporters, and hence, crossing with wild rice can be used to improve NUpE [66,243]. Using natural variation is especially valuable for trees, where obtaining transgenic plants and, even more so, their subsequent studies, are complex, lengthy and expensive. As with lignin modification [191], the use of natural genetic diversity for transgenic manipulations or in marker-mediated and genomic selection [244] is a promising strategy for improving the NUE in trees. Progress in this area will help protect the environment, develop sustainable agriculture and ensure food security, especially in developing countries that cannot apply expensive N fertilizers in adequate quantities.

## Figures and Tables

**Table 1:** Manipulation of nitrogen uptake genes to improve nitrogen use efficiency in crops.

Gene	Gene Source	Promoter	Target Crop	Technology ^1^	Growth Condition	N Condition	Type of NUE ^2^	References
Nitrate transporters								
*NRT2.1*	rice	CaMV 35S	rice	over	hydroponic	2 × 2 N levels	-	[55]
*NAR2.1*	rice		rice	RNAi	hydroponic	3 N levels	-	[56]
					pot	n.d. ^3^		
*PTR9*	rice	CaMV 35S	rice	over	hydroponic	4 N levels	-	[57]
		Ubi-1		RNAi	field	6 N levels		
*PTR6*	rice	ubiquitin	rice	over	hydroponic	5 N levels	NUE	[30]
*NRT1.1B*	rice	CaMV 35S	rice	over	hydroponic	3 NO_3_ levels	NUE	[58]
		NRT1.1b			field	2 NO_3_ levels		
*NRT2.3*	tomato	CaMV 35S	tomato	over	hydroponic	3 NO_3_ levels	-	[59]
*NRT2.3a* or *NRT2.3b*	rice	CaMV 35S	rice	over	hydroponic	2 N levels	NUE	[60]
		ubiquitin			field	4 urea levels		
*NRT1.1a* or *NRT1.1b*	rice	ubiquitin	rice	over	hydroponic	6 N levels	-	[61]
*NRT2.1*	rice	ubiquitin	rice	over	field	3 urea levels	ANUE, PNUE	[62]
		NAR2.1						
*NAR2.1*	rice	NAR2.1	rice	over	hydroponic	3 N levels	ANUE, PNUE	[63]
					field	1 N level		
*NPF7.3* (*PTR6*)	rice	CaMV 35S	rice	over	hydroponic	9 N levels	NUE	[64]
		Ubi-1		RNAi	field	1 N level		
*NPF7.7-1* or *NPF7.7-2*	rice	CaMV 35S	rice	over	hydroponic	9 N levels	NUtE	[65]
		Ubi-1		RNAi	field	4 NH_4_NO_3_ levels		
*NPF6.1*	rice	NPF6.1	rice	over	field	2 N levels	-	[66]
				editing				
*NPF4.5*	rice	ubiquitin	rice	over	hydroponic	2 N levels	-	[67]
				editing	pot			
*NPF1.11*	potato	CaMV 35S	potato	over	pot	1 N level	-	[68]
*NAR2.1*	rice	CaMV 35S	rice	over	field	2 N levels	ANUE, PNUE	[69]
*NRT2.3a*				stacking				
*NAR2.1*+ *NRT2.3a*								
*NC4N* (chimer.)	Arabidopsis	NRT1.7	rice	over	hydroponic	n.d.	NUE	[70]
					field	1 N level		
Ammonium transporters								
*AMT1.1*	rice	ubiquitin	rice	over	hydroponic	2 NH_4_ levels	-	[71]
*AMT1-1*	rice	ubiquitin	rice	over	hydroponic	3 N levels	-	[72]
*AMT1;1*	rice	ubiquitin	rice	over	hydroponic	6 NH_4_ levels	-	[73]
*AMT1-3*	rice	CaMV 35S	rice	over	hydroponic	4 NH_4_NO_3_ levels	-	[74]
					pot	4 N levels		
*AMT1;1a*	maize	Ubi-1	maize	over	hydroponic	2 NH_4_ levels		[75]

^1^ over = overexpression, RNAi = RNA interference, editing = CRISPR/Cas9; ^2^ (-) = no NUE calculation; ^3^ n.d. = no data.

**Table 2:** Manipulation of nitrogen assimilation genes to improve nitrogen use efficiency in crops.

Gene	Gene Source	Promoter	Target Crop	Technology	Growth Condition	N Condition	Type of NUE	References
Nitrate and nitrite reductases								
*nia*	tobacco	CaMV 35S	lettuce	over	pot	n.d.	-	[76]
*Nia2*	tobacco	CaMV 35S	potato	over	pot	2 NH_4_ + NO_3_ levels	-	[77]
*Nia2*	tobacco	CaMV 35S	potato	over	field	1 NH_4_ + NO_3_ level	-	[78]
*Nia2*	tobacco	CaMV 35S	potato	over	hydroponic	1 NO_3_ level	-	[79]
*Nia2*	tobacco	CaMV 35S	lettuce	over	pot	3 NO_3_ levels	-	[80]
*nia*	tobacco	CaMV 35S	wheat	over	pot	1 NO_3_ level	-	[81]
*NiR*	Arabidopsis	CERV	tobacco	over	hydroponic	2 NO_3_ levels	-	[82]
*Nia2* (2 variants)	tobacco	CaMV 35S	tobacco	over	hydroponic	3 NO_3_ levels	-	[83]
*GS1*	tobacco	CaMV 35S			field	n.d.		
*GOGAT*	Arabidopsis	CaMV 35S						
*ICDH*	tobacco	CaMV 35S						
*NR2*	rice	NR2	rice	over	field	1 NO_3_ level	NUE	[84]
		Ubi		RNAi				
*Nia2*	tobacco	CaMV 35S	tobacco	over	hydroponic	2 NO_3_ levels	-	[85]
					field	2 N levels		
Glutamine synthetase								
*GS15*	soybean	CaMV 35S	*Lotus corniculatus*	over	hydroponic	2 NH_4_ levels	-	[86]
*GS15*	soybean	rolD	*Lotus japonicus*	over	hydroponic	1 NO_3_ level	-	[87]
*GS1*	bean	rbcS	*wheat*	over	pot	n.d.	-	[88]
*GS* (cytosolic)	*Lotus japonicus*	LBC3	*Lotus japonicus*	antisense	hydroponic	2 N levels	-	[89]
*GS15*	soybean	CaMV 35S	pea	over	hydroponic	4 NO_3_ levels		[90]
		LBC3						
		rolD						
*GS1*	alfalfa	CaMV 35S	*Lotus japonicus*	over	pot	2 N levels		[91]
*GS15*	soybean	CaMV 35S	pea	over	hydroponic	4 NH_4_ levels		[92]
		LBC3						
		rolD						
*Gln1-3*	maize	CsVMV	maize	over	hydroponic	1 NO_3_ level	-	[93]
					pot	1 NO_3_ level		
*GS1;1*	rice	CaMV 35S	rice	over	hydroponic	2 NH_4_NO_3_ levels	-	[94]
*GS1;2*					field	1 N level		
*glnA*	*E. coli*	CaMV 35S						
*GS1;2*	rice	ubiquitin	rice	over	pot	3 N levels	NUtE	[95]
*GS1;1*	rice	CaMV 35S	rice	over	hydroponic	4 NH_4_NO_3_ levels	-	[96]
*Gln1*	sorghum	ubiquitin	sorghum	over	pot	2 N levels	-	[97]
*GS2*	Chinese cabbage	CaMV 35S	Chinese cabbage	over	hydroponic	7 N levels	-	[98]
*GS2*	wheat		wheat	over	field	2 urea levels		[99]
*GS1-1*	barley	GS1-1	barley	over	pot	3 NH_4_NO_3_ levels	NUE	[100]
*GS* *SPS*	soybean maize	CaMV 35S	alfalfa	over	pot	n.d.	-	[101]
*GS1.1*		NA	wheat	editing	hydroponic	3 N levels	NTE	[102]
					field	2 urea levels		
*Gln1-3* (2 copies)	maize	CsVMV + Rbcs	maize	over	field	1 urea level	-	[103]
Glutamate synthase								
*NADH-GOGAT*	rice	NADH-GOGAT	rice	over	hydroponic	n.d.	-	[104]
*AMT1;2*	rice	NA	rice	T-DNA tagging +	outdoor	1 N level	NUE	[105]
*GOGAT1*				crossing	field	1 N level		
*NADH-GOGAT*	wheat	actin1	maize	over	greenhouse	1 NH_4_ + NO_3_ level	-	[106]
*GOGAT* + *IDH*	sorghum	CsVMV		stacking				
*GOGAT* + *IDH* + *GDH*	maize	actin1						
*GOGAT* + *IDH* + *GDH* + *GS1.3*	maize	CsVMV or RbcS						
Glutamate dehydrogenase								
*GDH*	*E. coli*	ubiqutin	maize	over	field	3 NH_4_NO_3_ levels	-	[107]
*GDH*	*Aspergillus niger*	CaMV 35S	rice	over	hydroponic	2 NH_4_ levels	-	[108]
					field	1 N level		
*GDH*	*Aspergillus nidulans*	CaMV 35S	potato	over	pot	2 N levels	NUE	[109]
*GDH*	*Sclerotinia sclerotiorum*	ubiqutin	rice	over	hydroponic	3 NH_4_NO_3_ levels	-	[110]
*GDH*	*Pleurotus cystidiosus*	ubiqutin	rice	over	hydroponic	3 NH_4_ levels	-	[111]
					field	3 urea levels		
*GDH*	*Cylindrocarpon ehrenbergii*	ubiqutin	rice	over	hydroponic	3 NH_4_ levels	-	[112]
					field	2 urea levels		
*GDH*	*Eurotium cheralieri*	ubiqutin	rice	over	hydroponic	2 NH_4_NO_3_ levels	-	[113]
					field	3 N levels		
*GDH*	*Trichurus*	ubiqutin	rice	over	hydroponic	3 NH_4_ levels	-	[114]
					field	4 urea levels		

**Table 3:** Manipulation of nitrogen remobilization and translocation genes in crops.

Gene	Gene Source	Promoter	Target Crop	Technology	Growth Condition	N Condition	Type of NUE	References
Asparagine synthetase								
*asnA*	*E. coli*	rbcS	*Lotus corniculatus*	over	pot	n.d.	-	[115]
		plastocyanin						
*asnA*	*E. coli*	CaMV 35S	oilseed rape	over	pot	2 NO_3_ levels	-	[116]
*asnA*	*E. coli*	MAC	lettuce	over	pot	1 NH_4_ + NO_3_ level	-	[117]
*asnA*	*E. coli*	Pcpea	tomato	over	hydroponic	2 NH_4_ + NO_3_ levels	-	[118]
*ASN1*	rice	NA	rice	editing	hydroponic	2 NH_4_ levels	-	[119]
*ASN1*	rice	ubiqutin	rice	over	field	1 N level	-	[120]
Alanine aminotransferase								
*AlaAT*	barley	btg26	canola	over	hydroponic	1 NH_4_NO_3_ levels	-	[121]
		CaMV 35S			field	4 N levels		
*AlaAT*	barley	Ant1	rice	over	hydroponic	NH_4_ or NO_3_	-	[122]
*AlaAT*	barley	Ant1	rice	over	hydroponic	3 NH_4_ levels	NUpE	[123]
*AlaAT*	barley	Ant1	sugarcane	over	hydroponic	4 N levels	NUpE, NUtE, NUE	[124]
*AlaAT*	barley	Ant1	rice	over	hydroponic	2 NH_4_ levels		[125]
					field	3 N levels		
*AlaAT*	barley	Ant1	wheat	over	hydroponic	2 NO_3_ levels	-	[126]
		UBI4	sorghum		field	2 N levels		
*AlaAT*	cucumber	Ant1	rice	over	pot	3 N levels	NUpE, ANUE	[127]
*AlaAT*	barley	Ant1	rice	over	hydroponic (rice)	2 NH_4_ + NO_3_ levels	-	[128]
			barley		pot (all)	1 or 2 N levels		
			wheat		field (rice)	1 urea level		
Aspartate aminotransferase								
*AAT1*, *AAT2*, *AAT3*	rice	CaMV 35S	rice	over	hydroponic	1 N level	-	[129]
*AAT*	*E. coli*	CaMV 35S			field	n.d.		
*AAT*	alfalfa	btg26	canola	over	hydroponic	1 N level	-	[44]
					pot	2 urea levels		
Amino acid transporters								
*AAP1*	potato	CaMV 35S	potato	antisense	pot	n.d.	-	[130]
*AAP1*	*Vicia faba*	LeB4	*Vicia narbonensis*	over	pot	1 NH_4_ + NO_3_ level	-	[131]
			pea					
*AAP1*	*Vicia faba*	LeB4	pea	over	pot	n.d.	-	[132]
					field	n.d.		
*MMP1*	yeast	AAP1	pea	over	pot	1 N level	-	[133]
*AAP6a*	rice	CaMV 35S	rice	over	field	n.d.	-	[134]
				RNAi				
*AAP1*	pea	AAP1	pea	over	pot	1 N level	-	[135]
*AAP1*	pea	AAP1	pea	over	pot	3 NH_4_NO_3_ levels	NUpE, NUtE, NUE	[136]
*AAP3*	rice	CaMV 35S	rice	over	hydroponic	5 NH_4_NO_3_ levels	-	[137]
		Ubi-1		RNAi	field	n.d.		
				editing				
*AAP5*	rice	CaMV 35S	rice	over	hydroponic	n.d.	-	[138]
		Ubi-1		RNAi	field	n.d.		
				editing				
*LHT1*	rice	NA	rice	editing	field	n.d.	-	[139]
*LHT1*	rice	NA	rice	editing	field	1 N level	NUpE, NUtE	[140]
*AAP1*	rice	CaMV 35S	rice	over	hydroponic	3 NH_4_NO_3_ levels	-	[141]
		Ubi-1		RNAi	field	n.d.		
				editing				
*AAP4a* or *4b*	rice	CaMV 35S	rice	over	hydroponic	1 NH_4_NO_3_ level	-	[142]
*AAP4*		Ubi-1		RNAi	field	n.d.		
				editing				
*AAP13*	wheat	ub. or end.-sp.	wheat	over	pot	n.d.	-	[143]
		ubiquitin		RNAi				
*AAP1*	pea	CaMV 35S	pea	over + crossing	pot	1 N level	-	[144]
*SUT1*	pea	AAP1						

**Table 4:** Manipulation of transcription factors to improve nitrogen use efficiency in crops.

Gene	Gene Source	Promoter	Target Crop	Technology	Growth Condition	N Condition	Type of NUE	References
*CPK12*	rice	NA	rice	over	hydroponic	2 NH_4_NO_3_ levels	-	[145]
*Dof1*	maize	Ubi-1	rice	over	hydroponic	6 N levels	-	[146]
*NAC2-5A*	wheat	ubiquitin	wheat	over	hydroponic	2 NO_3_ levels	-	[147]
					pot	3 NO_3_ levels		
					field	2 urea levels		
*NAC-S*	wheat	ubiquitin	wheat	over	pot	n.d.	-	[148]
*Dof1*	maize	UBI4	wheat	over	hydroponic	2 NO_3_ levels	NUE	[149]
		rbcS1	sorghum		field	2 N levels		
*ESL4*	rice	NA	rice	over	greenhouse	4 N levels	NUE	[150]
					field	4 urea levels		
*GRF4*	rice	actin	rice	over	hydroponic	4 NH_4_NO_3_ levels	-	[151]
		GRF4	wheat	RNAi	field	1 urea level		
		CaMV 35S		editing				
*nac7*	maize	ubiquitin	maize	RNAi	pot	1 NO_3_ level	-	[152]
					field	n.d.		
*bZIP60*	wheat	ubiquitin	wheat	over	hydroponic	1 NO_3_ level	-	[153]
*GOGAT*				RNAi	field	1 urea level		
*CDF3*	Arabidopsis	CaMV 35S	tomato	over	hydroponic	2 NO_3_ levels	NUE	[154]
					pot	2 NO_3_ levels		
*NLP1*	rice	NLP1	rice	over	hydroponic	5 N levels	-	[155]
				editing	field	3 urea levels		
*myb61*	rice	NA	rice	editing	field	3 urea levels	NUE	[156]
*grf4*								
*NLP4*	rice	actin	rice	over	hydroponic	3 NO_3_ + NH_4_ levels	-	[157]
				editing	field	3 urea levels		

## Data Availability

The study did not report any data.
